# *Archigetes* Leuckart, 1878 (Cestoda, Caryophyllidea): diversity of enigmatic fish tapeworms with monoxenic life cycles

**DOI:** 10.1051/parasite/2022002

**Published:** 2022-02-09

**Authors:** Dalibor Uhrovič, Mikuláš Oros, Olena Kudlai, Roman Kuchta, Tomáš Scholz

**Affiliations:** 1 Institute of Parasitology, Slovak Academy of Sciences Hlinkova 3 040 10 Košice Slovak Republic; 2 University of Veterinary Medicine and Pharmacy in Košice Košice Slovak Republic; 3 Institute of Ecology, Nature Research Centre Akademijos 2 08412 Vilnius Lithuania; 4 Institute of Parasitology, Biology Centre of the Czech Academy of Sciences Branišovská 31 370 05 České Budějovice Czech Republic

**Keywords:** Species diversity, Eucestoda, Fish, Catostomidae, Ictiobinae, *lsr*DNA, Comparative morphology, Molecular prospecting, SEM, Histology, Nearctic Region

## Abstract

The caryophyllidean genus *Archigetes* Leuckart, 1878 is unique among all tapeworms in that its species can mature in invertebrate hosts (Oligochaeta), i.e., have a monoxenic (direct) life cycle. All five species were described as progenetic plerocercoids in oligochaetes and two of them also as adults from cypriniform fishes. Two species, *A. sieboldi* Leuckart, 1878 and *A. iowensis* Calentine, 1962, were found in North America in non-native common carp (*Cyprinus carpio*). A molecular study of caryophyllideans from the southern United States has revealed the occurrence of three new species in native freshwater fishes (Catostomidae, Ictiobinae): *Archigetes loculotruncatus* n. sp. from *Ictiobus bubalus*, *I. niger* and *Carpiodes cyprinus* is the largest representative of the genus and differs by a loculotruncate scolex. *Archigetes megacephalus* n. sp. from *Ictiobus niger*, *I. bubalus* and *I. cyprinellus* is characterised by a prominent, bothrioloculodiscate scolex. *Archigetes vadosus* n. sp. from *I. bubalus* is typified by a globular scolex with very shallow loculi; it differs from the closely related *A. sieboldi* in the shape of the body, with a distinct neck region and a scolex wider than the remaining body. *Archigetes iowensis* Calentine, 1962 becomes a junior synonym of *Paraglaridacris limnodrili* (Yamaguti, 1934). The generic diagnosis of *Archigetes* is amended and a key to identification of North American taxa is provided. Species of *Archigetes* and *Paraglaridacris* differ from each other most conspicuously in the structure of the ovary, which is follicular in *Archigetes* versus compact in *Paraglaridacris*.

## Abstract (fr)

Le genre *Archigetes* Leuckart, 1878 (Caryophyllidea) est unique parmi les cestodes en ce sens que ses espèces peuvent atteindre la maturité chez des hôtes invertébrés (Oligochaeta), c’est-à-dire avoir un cycle de vie monoxénique (direct). Les cinq espèces ont été décrites à partir de plérocercoïdes progénétiques chez des oligochètes et deux d’entre elles aussi à partir d’adultes chez des poissons cypriniformes. Deux espèces, *A. sieboldi* Leuckart, 1878 et *A. iowensis* Calentine, 1962, ont été trouvées en Amérique du Nord chez des carpes communes (*Cyprinus carpio*), non indigènes. Une étude moléculaire des caryophyllidés du sud des États-Unis a révélé la présence de trois nouvelles espèces chez les poissons d’eau douce indigènes (Catostomidae, Ictiobinae). *Archigetes loculotruncatus* n. sp. d’*Ictiobus bubalus*, *I. niger* et *Carpiodes cyprinus* est le plus grand représentant du genre et est caractérisé par un scolex loculotronqué. *Archigetes megacephalus* n. sp. d’*Ictiobus niger*, *I. bubalus* et *I. cyprinellus* est caractérisé par un scolex proéminent et bothrioloculodisqué. *Archigetes vadosus* n. sp. d’*I. bubalus* est caractérisé par un scolex globulaire avec des loges très peu profondes, et diffère d’*A. sieboldi*, étroitement apparenté, par la forme du corps, avec une région du cou distincte et un scolex plus large que le reste du corps. *Archigetes iowensis* Calentine, 1962 devient un synonyme plus récent de *Paraglaridacris limnodrili* (Yamaguti, 1934). Le diagnostic générique d’*Archigetes* est modifié et une clé d’identification des taxons nord-américains est fournie. Les espèces d’*Archigetes* et de *Paraglaridacris* diffèrent de la manière la plus visible les unes des autres par la structure de l’ovaire, qui est folliculaire chez *Archigetes* par opposition à compact chez *Paraglaridacris*.

## Introduction

The members of the genus *Archigetes* Leuckart, 1878 are extraordinary among tapeworms due to their direct (monoxenic) life cycle, i.e., the lack of an intermediate host, with sexual maturation in the invertebrate host. Since its erection by Leuckart [25], the genus has been the subject of intense speculation about the origin of the tapeworm life cycle [28, 29]. The monoxenic life cycle, with the production of eggs by progenetic plerocercoids in the body cavity of oligochaetes, and excluding fish as a typical caryophyllidean definitive host, was considered by some authors to be an ancestral type of the cestode life cycle, assuming that invertebrates were primary hosts of the most ancient tapeworms [1, 5, 11, 52].

This monoxenic cycle was found in all five species of the genus *Archigetes* recognised as valid [5, 19]. Accidentally infected freshwater teleosts most likely served as a postcyclic host, which may have resulted in the life cycle being extended to two host, i.e., heteroxenic (indirect) life cycle [11]. An alternative view is that some *Archigetes* species evolved by progenesis, i.e., early development of the reproductive system leading to sexual maturity (including the production of eggs) in a larval stage [36]. This scenario is supported by some morphological and developmental data [41, 42].

Molecular data have clearly shown that *Archigetes* is neither the most basal lineage within all cestodes nor within the order Caryophyllidea [36, 50]. This means that sexual maturity in the invertebrate host is unlikely to have an impact on understanding of the protocestode state [36]. In contrast, these molecular phylogenetic studies show that at least three *Archigetes* species are members of the most recently diverging caryophyllidean lineage, which comprises almost entirely Nearctic caryophyllideans [50]. The available data thus support the hypothesis that the monoxenic life cycle of *Archigetes* is a secondary abbreviation of the developmental cycle, similar to some spathebothriideans, such as *Cyathocephalus truncatus* (Pallas, 1781), *Diplocotyle olrikii* Krabbe, 1874 or *Spathebothrium simplex* Linton, 1922, whose maturation and egg production may occur in gammarid amphipods as their intermediate host [20, 35, 46].

In addition to controversies over the position of *Archigetes* and the evolutionary origin of its monoxenic life cycle, there is much confusion about the species composition of the genus. Kennedy [19] considered the following five species as valid: *A. sieboldi* Leuckart, 1878 (type species); *A. brachyurus* Mrázek, 1908; *A. cryptobothrius* Wisniewski, 1928; *A. limnodrili* (Yamaguti, 1934) Kennedy, 1965; and *A. iowensis*. However, no taxonomical revision based on properly fixed material and molecular data has been carried out. Moreover, the taxonomic position of some caryophyllideans placed in the genera *Biacetabulum* Hunter, 1927, *Brachyurus* Szidat, 1938 and *Paraglaridacris* Janiszewska, 1950 is also problematic, as they morphologically resemble the species of *Archigetes*.

The genus *Archigetes* was first reported from North America by Ward [57] who found unidentified tapeworms resembling those of *Archigetes* spp. known from Europe in a fish from the Illinois River at Havana. The genus was for a long time represented in North America by only two species, *A. iowensis* Calentine, 1962, described from non-native common carp (*Cyprinus carpio* L.) and *Limnodrilus hoffmeisteri* Claparède [5], and *A. sieboldi*, reported from the same hosts [4]. Recently, Scholz and Pérez-Ponce de León [49] reported three morphotypes of *Archigetes* tapeworms, which may represent putative new species, from eastern shiners, *Notropis* spp. (Cypriniformes: Leuciscidae), and silverside, *Chirostoma humboldtianum* (Valenciennes) (Atheriniformes: Atherinidae) in the Nearctic part of Mexico. The latter fish is the first representative of the order Atheriniformes that has been reported as the host of caryophyllidean tapeworms [49].

Recent studies on North American caryophyllideans have revealed high diversity of these tapeworms, previously only partly described. Two new genera were erected and nine new species of the genera *Biacetabulum* Hunter, 1927, *Promonobothrium* Mackiewicz, 1968 and *Isoglaridacris* Mackiewicz, 1965 described [37, 39, 40, 49, 54–56]. In the present paper, three new species of the enigmatic *Archigetes* are described from catostomid fishes from the southern United States (USA). In addition, species diversity of the genus is discussed together with the assessment of their phylogenetic relationships. The diagnosis of *Archigetes* is amended and a key to identification of North American taxa, including *A. iowensis* transferred to *Paraglaridacris*, is provided. Taxonomic status of morphologically similar species of the latter genus, which may also mature in oligochaetes, is discussed based on material from North America (USA), Asia (Japan) and Europe (Czech Republic, Russia and Slovakia).

## Materials and methods

The specimens studied were newly collected by the present authors and their collaborators in Mississippi (USA), in 2012 and 2019. In addition, specimens from the private collection of the late John S. Mackiewicz (USA) donated to the senior author (T.S.) and vouchers deposited in the Helminthological Collection of the Institute of Parasitology, Biology Centre of the Czech Academy of Sciences, České Budějovice (IPCAS) were examined.

Newly collected tapeworms were obtained from the intestines of freshly killed fish; they were rinsed with saline and fixed in hot, nearly boiling, 4% formaldehyde for morphological studies [38]. Some worms were fixed completely in 96% molecular-grade ethanol or in hot saline and then placed in 80% molecular-grade ethanol for DNA sequencing (see below). For light microscopy, specimens were stained in Mayer’s carmine, dehydrated in an ethanol series, cleared with clove oil (eugenol), and mounted in Canada balsam. Line drawings were made using a Leica DM 5000B light microscope (Leica Microsystems, Wetzlar, Germany). For scanning electron microscopy, selected specimens were examined using a Jeol I AM 6510LV and a Jeol JSEM 7401F electron microscope (Jeol Ltd., Tokyo, Japan) according to the procedure described by Oros et al. [38].

The phylogenetic relationships of the studied tapeworms were assessed based on the partial (D1–D3 region) nuclear ribosomal large subunit rRNA gene (*lsr*DNA) sequences. A list of sequenced samples and sequences used in the phylogenetic analysis is provided in Table 1. Genomic DNA was isolated using the Monarch Genomic DNA Purification Kit (New England Biolabs, Inc., USA), following the manufacturer’s instructions. A 1420 nucleotide (nt) long fragment of the 28S rRNA gene was amplified following the protocol described by Brabec et al. [3] or Scholz et al. [51]. An ExoSAP-IT PCR Cleanup enzymatic kit from Thermo Fisher Scientific, Inc. (Waltham, MA, USA) was used to purify the PCR products, following the manufacturer’s protocol. PCR amplicons were thereafter sequenced from both strands using the PCR primers and additional internal sequencing primer 300F and ECD2 [26]. Sequences were assembled and edited using Geneious version 11 (Biomatters, Auckland, New Zealand). The new *lsr*DNA sequences were aligned with the sequences of *Archigetes* spp. and *Biacetabulum* spp. available in GenBank using ClustalW implemented in Geneious ver. 11 (Biomatters, Auckland, New Zealand). The length of the final alignment was 1329 nucleotides (nt).

**Table 1:** Host, geographical origin and GenBank accession data for taxa included in the phylogenetic analysis.

Species	Host	Locality	Morphological voucher^a^	GenBank ID	Source
*Archigetes loculotruncatus* n. sp.	*Ictiobus bubalus*	Mississippi, USA	US 260d/PBI-464	MW027502	Scholz et al. [50]
*Archigetes megacephalus* n. sp.	*Ictiobus cyprinellus*	Mississippi, USA	US 242a/PBI-462	MW027493	Scholz et al. [50]
*Archigetes megacephalus* n. sp.	*Ictiobus niger*	Mississippi, USA	US 244b/PBI-417	MW027494	Scholz et al. [50]
*Archigetes megacephalus* n. sp.	*Ictiobus niger*	Mississippi, USA	US244b	OM103263	Present study
*Archigetes megacephalus* n. sp.	*Ictiobus niger*	Mississippi, USA	US244b-2	OM103264	Present study
*Archigetes megacephalus* n. sp.	*Ictiobus bubalus*	Mississippi, USA	US260b	OM103265	Present study
*Archigetes vadosus* n. sp.	*Ictiobus bubalus*	Mississippi, USA	US828a	OM103266	Present study
*Archigetes vadosus* n. sp.	*Ictiobus bubalus*	Mississippi, USA	US831d	OM103267	Present study
*Archigetes vadosus* n. sp.	*Ictiobus bubalus*	Mississippi, USA	US862b	OM103268	Present study
*Archigetes vadosus* n. sp.	*Ictiobus bubalus*	Mississippi, USA	US862c	OM103269	Present study
*Archigetes vadosus* n. sp.	*Ictiobus bubalus*	Mississippi, USA	US831d-3	OM103270	Present study
*Archigetes vadosus* n. sp.	*Ictiobus bubalus*	Mississippi, USA	US831d-4	OM103271	Present study
*Archigetes sieboldi*	*Gobio gobio*	České Budějovice, Czech Republic	PBI-43/TS-09/155	MW027492	Scholz et al. [50]
*Archigetes sieboldi*	*Gnathopogon elongatus*	Japan	–	EU343736	Olson et al. [36]
*Biacetabulum giganteum*	*Ictiobus bubalus*	Oklahoma, USA	US 749	OK070765	Uhrovič et al. [55]
*Biacetabulum johni*	*Minytrema melanops*	Florida, USA	US 164a	OK070762	Uhrovič et al. [55]
*Biacetabulum isaureae*	*Moxostoma collapsum*	South Carolina, USA	US 272c	MZ031044	Uhrovič et al. [54]
*Biacetabulum longicollum*	*Hypentelium nigricans*	Tennessee, USA	US 406/1	OK070760	Uhrovič et al. [55]
*Biacetabulum macrocephalum*	*Catostomus commersonii*	New York, USA	US1053	OM103272	Present study
*Biacetabulum macrocephalum*	*Catostomus commersonii*	Wisconsin, USA	DP66/09b	MW027498	Scholz et al. [50]
*Biacetabulum magdae*	*Minytrema melanops*	Mississippi, USA	PBI-415/US 217b	MW027496	Scholz et al. [50]
*Biacetabulum overstreeti*	*Minytrema melanops*	Mississippi, USA	US 233b/PBI-416	MW027499	Scholz et al. [50]
*Biacetabulum* sp. 3	*Minytrema melanops*	Mississippi, USA	US 217b	OK070767	Uhrovič et al. [55]
*Biacetabulum* sp. 4	*Minytrema melanops*	Mississippi, USA	US 238/1	OK070768	Uhrovič et al. [55]
b *Hypocaryophyllaeus paratarius*	*Carpiodes cyprinus*	Wisconsin, USA	PBI-405/2	MW027507	Scholz et al. [50]
b *Isoglaridacris erraticus*	*Moxostoma macrolepidotum*	South Carolina, USA	PBI-467/US 281a	MW027510	Scholz et al. [50]
b *Pseudoglaridacris laruei*	*Catostomus commersonii*	Connecticut, USA	PBI-54/TS-09/294	MW027527	Scholz et al. [50]

a US – United States samples collected by the present authors in 2012 and 2019, by TJ. Fayton and C.T. McAllister in 2014 (US 406), and F. Reyda in 2021 (US 1053); PBI – Planetary Biodiversity Inventory numbers refer to samples used in a large-scale National Science Foundation-funded (NSF-PBI) study of tapeworms (see [50]). Data on individual samples sequenced can be found under a given PBI number at http://tapewormdb.uconn.edu/index.php/parasites/molecular_search/; TS – samples collected by T. Scholz in 2009; DP – acronym of De Pere, i.e., a sample collected by M. Oros in 2009 in Wisconsin.

b Outgroup.

Bayesian inference (BI) and maximum likelihood (ML) methods were used to assess phylogenetic relationships within the dataset. The best-fitting model for the analyses, GTR + I + G, was estimated using jModelTest 2.1.2 [10]. MrBayes software (ver. 3.2.3) [45] was used to perform the BI analysis. Markov chain Monte Carlo analyses were run for 10,000,000 generations, log-likelihood scores were plotted, and only the final 75% of trees were used to build the consensus tree. ML analysis was performed using PhyML version 3.0 [13] with nonparametric bootstrap validation based on 100 pseudoreplicates. FigTree ver. 1.4 software [44] was used to visualise the phylogenetic trees. To calculate genetic distances (uncorrected *p*-distance), a separate alignment including only sequences of *Archigetes* spp. (1356 nt) was used in MEGA ver. X [24].

In addition to IPCAS (see above), the studied specimens are deposited in the National Helminthological Collection of Mexico, Instituto de Biología, Universidad Nacional Autónoma de México, Mexico City, Mexico (CNHE), Harold W. Manter Laboratory, University of Nebraska, Lincoln, USA (HWML), and the National Museum of Natural History, Washington, D.C., USA (USNM). The terminology of the scolex morphology follows the proposal of Mackiewicz [30] and Oros et al. [39]. Terminology of microtriches follows Chervy [9]. The scientific and common names of fish hosts follow FishBase [12]. In descriptions, measurements are given in micrometres unless otherwise noted.

Host code numbers correspond to a unique individual fish host examined and small letters specify individual tapeworm(s) found in this host, i.e., correspond to morphological voucher(s). See Table 1.

## Results

### Molecular phylogeny

**Figure 1:**
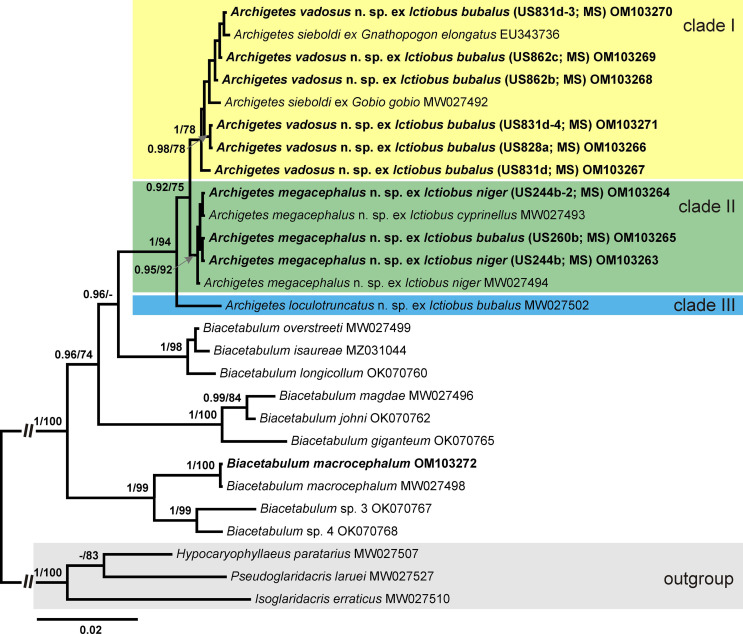
Bayesian phylogram of *lsr*DNA for (Cestoda: Caryophyllidea) Nodal support from Bayesian inference (BI) and maximum likelihood (ML) bootstrap support are indicated as BI/ML; values < 0.90 (BI) and < 70 (ML) are not shown. The scale bar indicates the expected number of substitutions per site. The newly generated sequences are highlighted in bold. Coloured rectangles indicate new species of *Archigetes* identified in this study Abbreviation: MS, Mississippi (country code for host field numbers). *Archigetes megacephalus* (MW027493 and MW027494) correspond to *Archigetes* sp. 2, and *Archigetes loculotruncatus* (MW027502) corresponds to *Archigetes* sp. 1 in Scholz et al. [50].

Molecular phylogenetic analysis, which included sequences of nine newly recovered isolates of *Archigetes* and sequences of this genus available in GenBank, revealed a well-supported monophyletic group (Fig. 1). *Archigetes* spp. were positioned in three clades. Clade I contained isolates of two morphologically distinct species, i.e., the type species of the genus, *A. sieboldi*, from Europe and Japan and a putative new species from *I*. *bubalus* collected in North America. The genetic divergence within this clade was 0–5 nt (0–0.37%). In contrast to conspicuous morphological differences (see below), the interspecific divergence between *A*. *sieboldi* and this new species was rather low and ranged between 1 and 5 nt (0.08–0.37%). Clades II and III contained isolates representing two species from *Ictiobus* spp. in North America that were recognised as species new to science. Sequences of Clade II were identical. The three new species are described below. The nucleotide divergence of sequences of each isolate is summarised in Supplementary Table S1.

### Species diversity of *Archigetes* Leuckart, 1878

Several taxa have been placed in *Archigetes*, but only the four following species were recently recognised as valid [48]: *A. sieboldi* Leuckart, 1878 (type species); *A. brachyurus* Mrázek, 1908 (both species found in oligochaetes and fish [43]); *A. cryptobothrius* Wisniewski, 1928 (known only from oligochaetes); and *A. iowensis* (species reported from oligochaetes and fish). Scholz and Oros [48] erroneously listed *Paraglaridacris limnodrili* (Yamaguti, 1934) Mackiewicz, 1994 also among valid species of *Archigetes*. The validity of two species, namely *A. brachyurus* and *A. cryptobothrius*, which have been reported only from Europe [19, 43], could not be critically assessed because of the lack of properly fixed material and molecular data.

*Archigetes sieboldi* and *A. iowensis* also occur in North America and their taxonomic status and biogeography are briefly discussed below. In addition to the three unidentified morphotypes of *Archigetes* recently reported from Mexico [49], another three new species found in catostomids from the southern USA are described below based on morphological and molecular data.

#### *Archigetes loculotruncatus* n. sp. (Figs. 2–5; Table 2)

Syn.: *Archigetes* sp. 1 of Scholz et al. [50]

**Figure 2:**
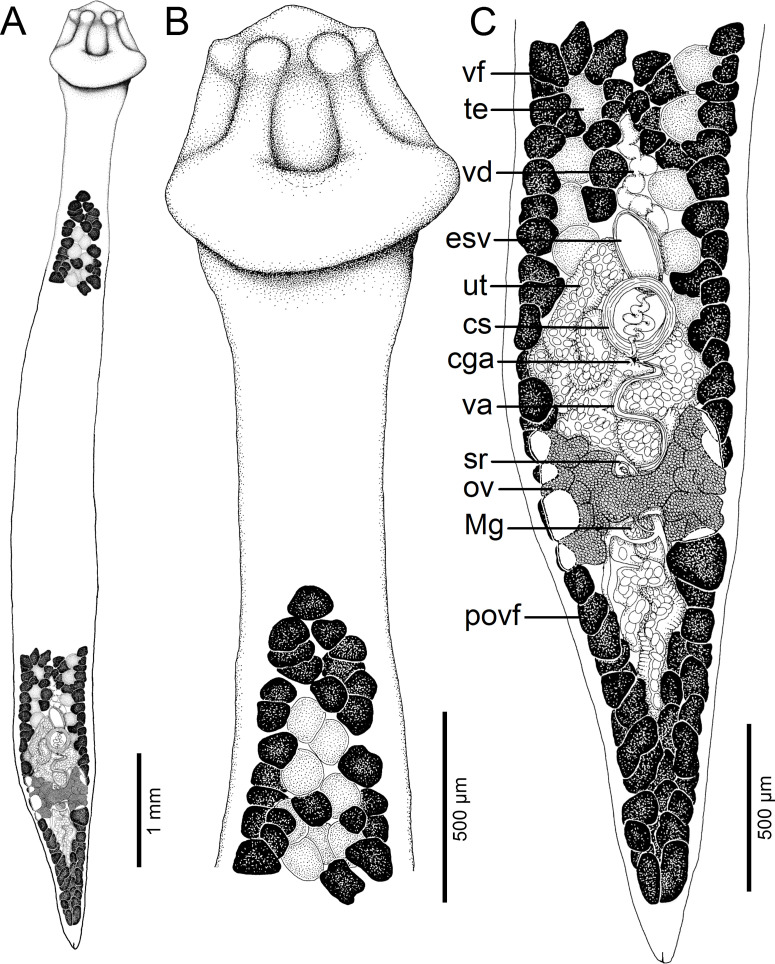
Line drawings of *Archigetes loculotruncatus* n. sp. from *Ictiobus bubalus* (Rafinesque), Chotard Lake near Vicksburg, Mississippi, USA (host field code US 260d, IPCAS C-903/1, PBI-464). A *–* total view (testes and vitelline follicles are not illustrated in the middle portion of the body), B *–* anterior part with scolex, C *–* posterior part. Abbreviations: cga – common genital atrium, cs – cirrus-sac, esv – external seminal vesicle, Mg – Mehlis’ gland, ov – ovary, povf – postovarian vitelline follicles, sr – *receptaculum seminis*, te *–* testes, ut – uterus, va – vagina, vd *– vas deferens*, vf *–* vitelline follicles.

**Table 2:** Comparative measurements of new species of *Archigetes* from ictiobine fish (Catostomidae) in Mississipppi, USA.

Species	*Archigetes loculotruncatus* n. sp.		*Archigetes megacephalus* n. sp.					*Archigetes vadosus* n. sp.
Host	Ictiobus bubalus*		*I. bubalus*					I. bubalus*
	*I. niger*		*I. cyprinellus*					
	*Carpiodes cyprinus*		I. niger*					
Character/no. of specimens	*n* = 10	(mean ± SD)	*n* = 10		(mean ± SD)		*n* = 7	(mean ± SD)
Body length (mm)	6.0–8.3	(7.2 ± 0.8)	2.6–4.8		(3.6 ± 0.7)		2.8–6.6	(4.4 ± 1.3)
Maximum width	523–757	(634 ± 72)	366–544		(480 ± 57)		380–567	(485 ± 73)
Width at cirrus sac-level	543–751	(608 ± 50)	364–535		(452 ± 59)		341–505	(416 ± 61)
Ratio of body width to body length	7–11%	(9 ± 1%)	10–17%		(14 ± 2%)		9–15%	(11 ± 2%)
Scolex length	629–787	(698 ± 63)	569–753		(649 ± 56)		466–690	(599 ± 82)
Width	622–954	(768 ± 131)	604–811		(715 ± 86)		473–704	(591 ± 94)
Ratio of scolex width to neck width	41–67%	(49 ± 7 %)	30–49%		(38 ± 6%)		50–63%	(56 ± 6%)
Ratio of scolex width to body width	95–157%	(122 ± 19%)	115–167%		(150 ± 18%)		113–132%	(122 ± 6%)
Ratio of scolex width to body length	9–13%	(11 ± 1%)	16–28%		(21 ± 4%)		11–18%	(14 ± 3%)
Neck length	716–1049	(781 ± 74)	99–637		(353 ± 214		163–407	(246 ± 119)
Width	331–416	(377 ± 38)	231–338		(269 ± 38)		246–398	(327 ± 52)
Ratio of neck length to body length	10–15%	(12 ± 2%)	4–16%		(9 ± 4%)		2–9%	(6 ± 2%)
Ratio of neck width to body width	50–65%	(59 ± 5%)	46–69%		(57 ± 9%)		59–80%	(68 ± 8%)
Testis size (length × width)	110–178 × 82–144 (*n* = 30)		83–154 × 77–116 (*n* = 30)			52–129 × 50–129 (*n* = 21)		
Number	72–145	(86 ± 14)	37–65	(53 ± 9)			76–149	(100 ± 26)
Number/500 μm of length of testicular area	9–22	(14 ± 5)	14–26	(18 ± 5)			13–38	(24 ± 9)
Distance from first vitelline follicles	106–366	(226 ± 85)	44–203	(115 ± 54)			47–267	(149 ± 26)
Distance from anterior extremity	1573–2004	(1658 ± 68)	727–1581	(1110 ± 301)			734–1132	(963 ± 156)
Testicular area length (mm)	2.9–4.6	(4.0 ± 0.6)	1.1–2.1	(1.5 ± 0.3)			1.3–3.4	(2.2 ± 0.7)
Extent in relation to the body width at CS level	19–29%	(23 ± 3%)	22–37%	(27 ± 5%)			17–31%	(23 ± 5%)
Ratio of testicular area length to total body length	49–57%	(54 ± 3%)	36–48%	(42 ± 3%)			42–60%	(50 ± 6%)
Cirrus-sac size (length × width)	207–254 × 195–238	124–166 × 123–172	146–178 × 113–165					
Ratio of cirrus-sac width to body width at CS level	31–41%	(36 ± 3%)	28–37%	(33 ± 3%)			32–43%	(50 ± 6%)
External seminal vesicle (length × width)	208–340 × 111–174	125–208 × 59–88	131–267 × 65–104					
Ratio of ESV length to CS length	64–124%	(90 ± 20%)	74–104%	(85 ± 10%)			62–111%	(85 ± 17%)
Distance of genital atrium from posterior end	1201–1801	(1390 ± 181)	611–1040	(840 ± 158)			652–1202	(890 ± 200)
Ovary width	381–557	(455 ± 46)	224–380	(308 ± 55)			238–340	(296 ± 40)
Length of ovarian wings	211–500	(395 ± 47)	204–308	(254 ± 41)			220–409	(294 ± 69)
Width of ovarian wings	89–161	(133 ± 18)	75–127	(96 ± 14)			62–117	(96 ± 16)
Ratio of length of ovarian wings to length of body	4–6%	(6 ± 1%)	6–9%	(8 ± 1%)			5–8%	(7 ± 1%)
Ratio of length of ovarian wings to length of uterine area	31–40%	(34 ± 3%)	23–40%	(35 ± 5%)			34–49%	(40 ± 6%)
Vitelline follicle size (length × width)	73–138 × 60–105 (*n* = 30)	63–101 × 39–88 (*n* = 30)	36–98 × 34–90 (*n* = 21)					
Distance of anteriormost vitelline follicles from anterior extremity	1061–1781	(1432 ± 88)	654–1380	(997 ± 270)			694–1028	(813 ± 122)
Length of uterine area	954–1456	(1202 ± 171)	545–896	(755 ± 130)			553–1018	(754 ± 156)
Ratio of uterine area to testicular area	28–34%	(31 ± 2%)	42–71%	(51 ± 8%)			25–42%	(35 ± 6%)
Ratio of uterine area to length of body	15–18%	(17 ± 1%)	18–27%	(21 ± 3%)			15–20%	(17 ± 2%)
Size of intrauterine eggs	46–52 × 26–35 (*n* = 40)		35–47 × 22–39 (*n* = 40)				40–52 × 25–35 (*n* = 28)	
Shape of field of postovarian vitelline follicles	V		V				V	

* Type host.

urn:lsid:zoobank.org:act:1C1DE51D-7DC2-41A6-9CCB-9AC1BC31089F

*Type host*: *Ictiobus bubalus* (Rafinesque), Smallmouth buffalo.

*Other host*: *Ictiobus niger* (Rafinesque), Black buffalo; *Carpiodes cyprinus* (Lesueur), Quillback (all Cypriniformes: Catostomidae, Ictiobinae).

*Site in host*: Anterior intestine.

*Type locality*: Chotard Lake near Vicksburg, Mississippi, USA (32.587867; −91.021317).

*Additional localities*: Pascagoula River, Benndale; Sunflower River, Indianola (all Mississippi, USA); Reelfoot Lake, Tennessee, USA.

*Type material*: Holotype from *I. bubalus* (host code No. US 260d) collected on 24 March 2012 (IPCAS C-903/1); three paratypes from *I. bubalus* (US 260d, US 257a) (IPCAS C-903/1); four paratypes from *I. niger* (US 244b) (HWML 216781; IPCAS C-903/2); two paratypes from *I. bubalus* (US 257a) (USNM 1661733).

*Material studied*: Four slides with eight whole-mounted specimens and five slides with sagittal and cross sections of another specimen from *I. bubalus* (US 257a and 260b – PBI464); five slides with nine specimens (one incomplete) and five slides with longitudinal and cross sections of another specimen from *I. niger* (US 244b); one slide with one whole-mounted specimen from *C. cyprinus* (host code No. US 262b), all specimens from Chotard Lake near Vicksburg, collected by the authors (R.K. and M.O.) on 23 and 24 March 2012; one slide with whole-mounted specimen from *C. cyprinus* (host code No. US 171a) from Pascagoula River, near Benndale, coll. by the same authors on 18 March 2012; one slide with whole-mounted specimen from *I. bubalus* (host code No. RF3/490), Reelfoot Lake, donated by J.S. Mackiewicz to T.S.; two slides with two immature specimens from *I. niger* (FR19_765), Sunflower River coll. by F. Reyda on 17 August 2019.

*Representative DNA sequences and phylogenetic relationships*: Sequences of one individual from *I. bubalus* (US 260d/PBI-464) in Mississippi were provided by Scholz et al. [50]: *ssr* DNA (GenBank Accession Nos. MW027441), *lsr* DNA (MW027502) and *rrnL* (MW027379); Scholz et al. [50]). This species differs most conspicuously from all other sequenced species in *lsr*DNA sequences by 16–20 nt (1.18–1.48%) (Supplementary Table S1).

*Etymology*: The species name *loculotruncatus* refers to the loculotruncate scolex, which is a unique characteristic of this new species among other members of the genus *Archigetes*.

**Figure 3:**
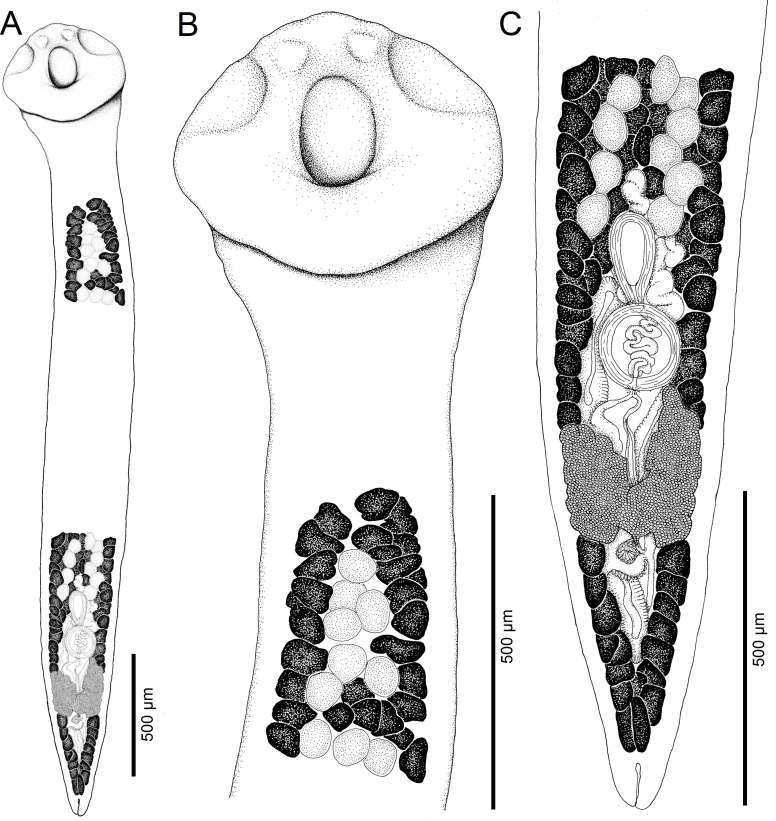
Line drawings of *Archigetes loculotruncatus* n. sp. from *Carpiodes cyprinus* (Lesueur), Chotard Lake near Vicksburg, Mississippi, USA (US 262b, IPCAS C-903/3). A *–* total view (testes and vitelline follicles are not illustrated in the middle portion of the body), B *–* anterior part with scolex, C *–* posterior part.

**Figure 4:**
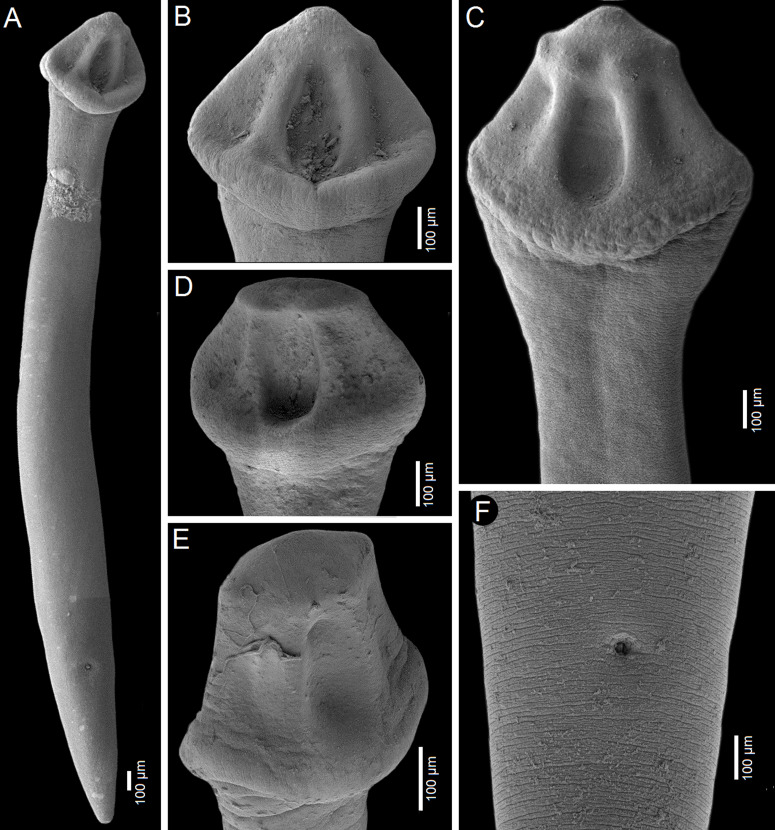
Scanning electron micrographs of *Archigetes loculotruncatus* n. sp. A, B, C, F – from *Ictiobus bubalus* (Rafinesque) (US 257a and US 260d, IPCAS C-903/1, PBI-464); D – from *I. niger* (Rafinesque, 1818) (US 244b, IPCAS C-903/2), Chotard Lake near Vicksburg, Mississippi, USA; E – from *I. niger* (Rafinesque, 1818) (FR19_765, IPCAS C-903/2), Sunflower River in Indianola, Mississippi, USA. A – total view, B–E – variability in scolex shape, F – posterior part with common genital atrium.

**Figure 5:**
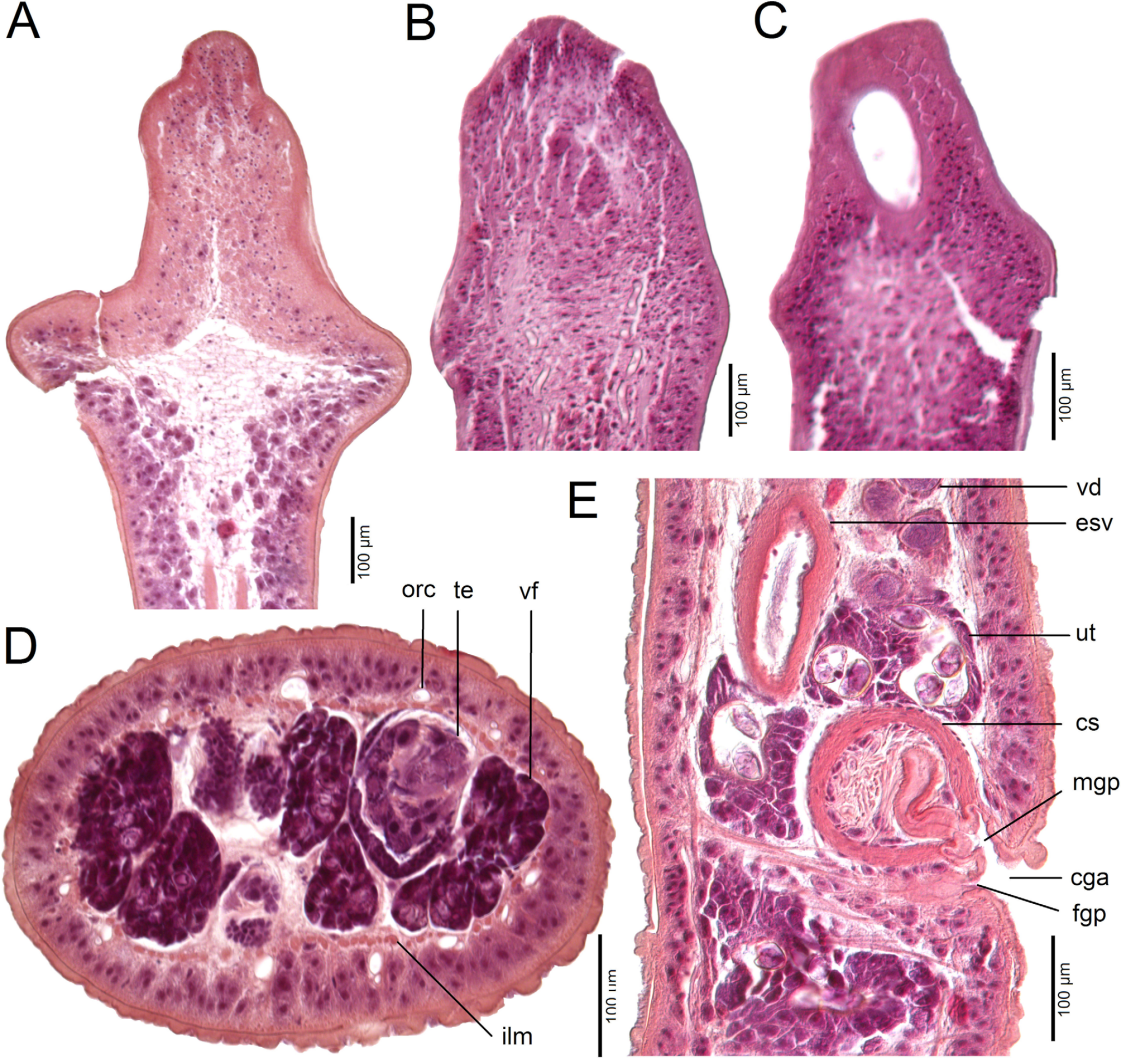
Histological section of *Archigetes loculotruncatus* n. sp. from *Ictiobus niger* (Rafinesque) (US 244b, IPCAS C-903/3), Chotard Lake near Vicksburg, Mississippi, USA. A – sagittal section of scolex; B, C – longitudinal sections of scolex; D – cross section of middle portion of body; E – sagittal section of genital pores. Abbreviations: cga – common genital atrium, cs – cirrus-sac, esv – external seminal vesicle, fgp – female gonopore, ilm – inner longitudinal muscles, mgp – male gonopore, orc – osmoregulatory canals, te *–* testes, ut – uterus, vd *– vas deferens*, vf *–* vitelline follicles.

*Description* (based on whole mounts of 21 specimens; for measurements – see Table 2): Caryophyllidea, Capingentidae *sensu* Scholz et al. [50]. Body elongate, with maximum width from mid-length to level of ovary, slightly tapering towards neck region anteriorly and sharply narrowing posterior to ovary towards pointed posterior end (Figs. 2A, 3A, 4A). Body covered with acicular fillitriches. Scolex loculotruncate (corresponding to scolex type illustrated in Fig. 5.3 by Mackiewicz [30]), wider than distinct, relatively long neck (Figs. 2A, 2B; 3A, 3B). Scolex with one median pair of ovoid acetabulum-like loculi, two pairs of shallower lateral loculi, and widely conical apical disc (Figs. 2B; 3B, 3C; 4A–4E; 5A–5C). Internal and external longitudinal musculature well-developed. Osmoregulatory canals in cortex, forming 12–14 pairs of narrow canals (Fig. 5D).

Testes medullary, subspherical to widely oval (Figs. 2B, 2C; 3B, 3C; 5D). Anterior-most testes begin posterior to anterior-most vitelline follicles. Posteriorly, testes reach to level of external seminal vesicle, rarely up to cirrus-sac (Figs. 2C, 3C). Cirrus-sac subspherical to spherical, thick-walled, situated anterior to ovary. External seminal vesicle ovoid, thick-walled (Figs. 2C, 3C). Male genital pore opens anterior to female gonopore to common genital atrium (Fig. 5E).

Ovary butterfly-shaped, with follicular lateral wings (Figs. 2C, 3C). Vagina tubular, sinuous, widened to form elongate, narrow seminal receptacle anterodorsal to ovarian isthmus, joins uterus to form uterovaginal canal opening close to but separate from male gonopore at bottom of distinct genital atrium (corresponding to Fig. 5.24 of Mackiewicz [30]) (Figs. 2C, 3C, 4F, 5E). Preovarian vitelline follicles numerous, in medullary parenchyma (Figs. 2B, 2C; 3B, 3C; 5D). Preovarian vitelline follicles may be partially connected with postovarian vitelline follicles by a few follicles situated irregularly alongside (laterodorsal to) ovarian wings (Fig. 2C). Postovarian vitelline follicles relatively numerous, forming V-shaped field (Fig. 2C; Table 2).

Uterus forms several loops, with single loop extending slightly anterior to cirrus-sac (Figs. 2C, 3C); uterine glands well-developed, absent only in distal and proximal parts of uterus. Eggs operculate, without fully formed oncosphere *in utero*.

#### Differential diagnosis

The new species is the largest member of the genus with a total body length of 6.0–8.3 mm *vs* 1.1–3.5 mm in the other five valid species. It also differs from all valid species of *Archigetes*, including the other two new species described below, in possessing a loculotruncate (Figs. 2B; 3B, 3C; 4A–4E; 5A–5C), rather than a bothrioloculodiscate or bulboloculate scolex as reported in other species (for types of scoleces – see Mackiewicz [30]).

The new species is also distinguished from the five previously known species by (i) a relatively long neck, whose length represents 10–15% of the total body length and which is absent or indistinct in the other species; (ii) a butterfly-shaped ovary in the new species *vs* dumb-bell-shaped ovary; (iii) an elongate seminal vesicle, which is longer than the diameter of the cirrus-sac in the new species *vs* spherical and smaller than the cirrus-sac in the other valid species.

#### Remarks

The new species is placed in the genus *Archigetes* based on morphological and molecular data (see Fig. 1 in Scholz et al. [50]), but differs from nominal species in having a distinct, long neck and a butterfly-shaped ovary. *Archigetes loculotruncatus* has been found in two species of *Ictiobus* and in *Carpiodes cyprinus*, i.e., suckers of the subfamily Ictiobinae. Most specimens were found in Chotard Lake, a former branch of the Mississippi River on the Mississippi-Louisiana state line. However, some specimens have also been found in the quillback from the Pascagoula River near Benndale, in southeastern Mississippi, in black buffalo from the Sunflower River in Indianola, east of the Mississippi River in central Mississippi, and smallmouth buffalo from Reelfoot Lake in Tennessee. This indicates a relatively large range for the new species in the southern USA.

#### *Archigetes megacephalus* n. sp. (Figs. 6–8; Table 2)

Syn.: *Archigetes* sp. 2 of Scholz et al. [50]

**Figure 6:**
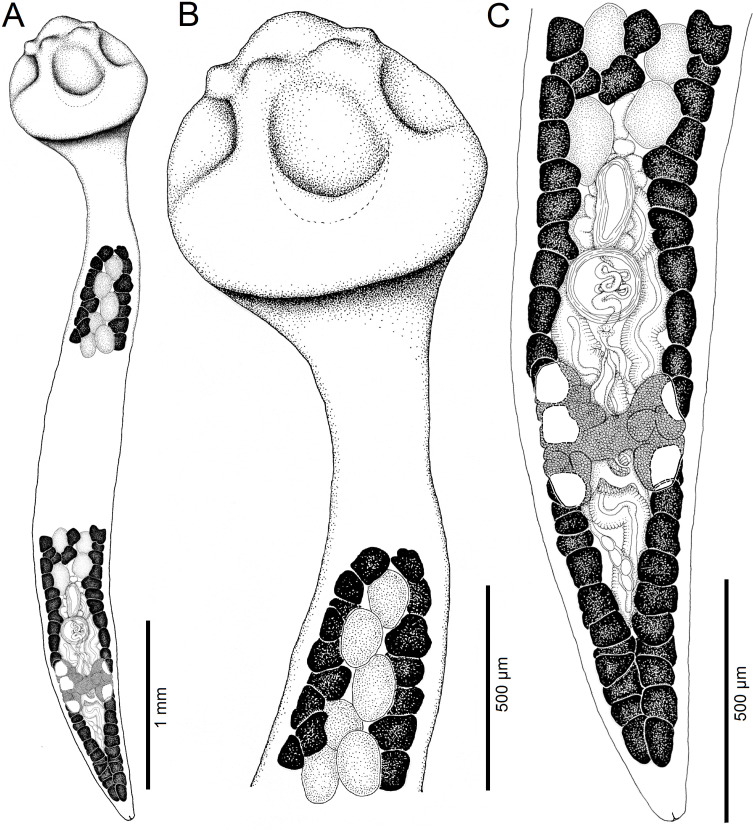
Line drawings of *Archigetes megacephalus* n. sp. from *Ictiobus niger* (Rafinesque), Chotard Lake near Vicksburg, Mississippi, USA (US 244b, IPCAS C-904/3, PBI-417). A *–* total view (testes and vitelline follicles are not illustrated in the middle portion of the body), B *–* anterior part with scolex, C *–* posterior part.

urn:lsid:zoobank.org:act:66185BB1-AF2D-456C-BA5E-7A83385207CE

*Type host*: *Ictiobus niger* (Rafinesque), Black buffalo.

*Other host*: *Ictiobus bubalus* (Rafinesque), Smallmouth buffalo; *Ictiobus cyprinellus* (Valenciennes), Bigmouth buffalo (all Cypriniformes: Catostomidae, Ictiobinae).

*Site in host*: Anterior intestine.

*Type locality*: Chotard Lake near Vicksburg, Mississippi, USA (32.587867; −91.021317).

*Additional localities*: Pascagoula River, Wilkerson’s Ferry launch (all Mississippi, USA), Reelfoot Lake, Tennessee, USA.

*Type material*: Holotype from *I. niger* collected on 23 March 2012 (host code No. US 244b) (IPCAS C-904/3); two paratypes from *I. niger* (US 244b) (IPCAS C-904/3; USNM 1661735); seven paratypes from *I. cyprinellus* (US 242a) (HWML 216782; IPCAS C-904/1; USNM 1661734); one paratype from *I. bubalus* (IPCAS C-904/2).

*Material studied*: One slide with one whole-mounted specimen from *I. bubalus* (US 257b), collected by the present authors (R.K. and M.O.) on 24 March 2012; two slides with two specimens from *I. bubalus* (DNA-02-267D), coll. by R.M. Overstreet and S.S. Curran in 2002; five slides with eight whole-mounted specimens and one slide with longitudinal sections of another specimen from *I. cyprinellus* (host code No. US 242a – PBI-462); three slides with four specimens from *I. niger* (US 244b – PBI-417), coll. authors (R.K. and M.O.) on 23 March 2012; all specimens from Chotard Lake near Vicksburg; two slides with five whole-mounted specimens from *I. bubalus* (host code No. RF3/490), Reelfoot Lake, donated by J.S. Mackiewicz to T.S.

*Representative DNA sequences and phylogenetic relationships*: The *lsr*DNA sequences of one individual from *I. bubalus* (US 260b – OM103265), one from *I. cyprinellus* (US 242a/PBI-462 – MW027493), and three from *I. niger* (US 244b/PBI-417 – MW027494; OM103263; OM103264), all from Chotard Lake. Three novel sequences and two sequences retrieved from GenBank were identical. They differed from the sequence of *Archigetes loculotruncatus* n. sp. by 16 nt (1.18%) (Supplementary Table S1).

*Etymology*: The species name, *mega* – very big; *cephalus* – head or scolex, refers to a conspicuously large scolex, the width of which considerably exceeds that of the neck.

**Figure 7:**
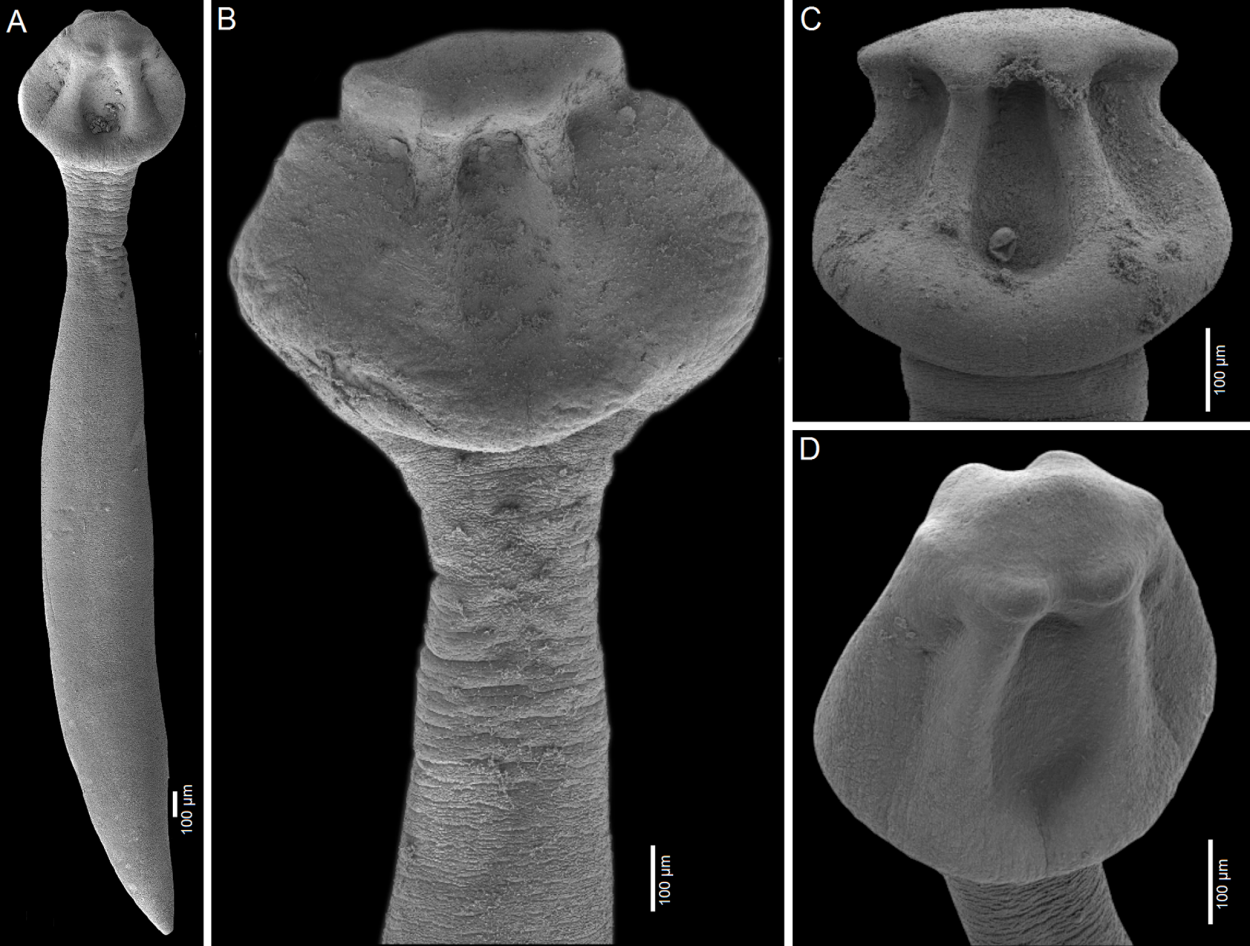
Scanning electron micrographs of *Archigetes megacephalus* n. sp. A, B – from *Ictiobus bubalus* (Rafinesque) (US 257a and US 260b, IPCAS C-904/2); C – from *I. cyprinellus* (Valenciennes, 1844) (US 242a, IPCAS C-904/1, PBI-462); D – from *I. niger* (Rafinesque) (US 244b, IPCAS C-904/3, PBI-417), Chotard Lake near Vicksburg, Mississippi, USA. A – total view, B – detail of scolex, C, E – variability in scolex shape.

**Figure 8:**
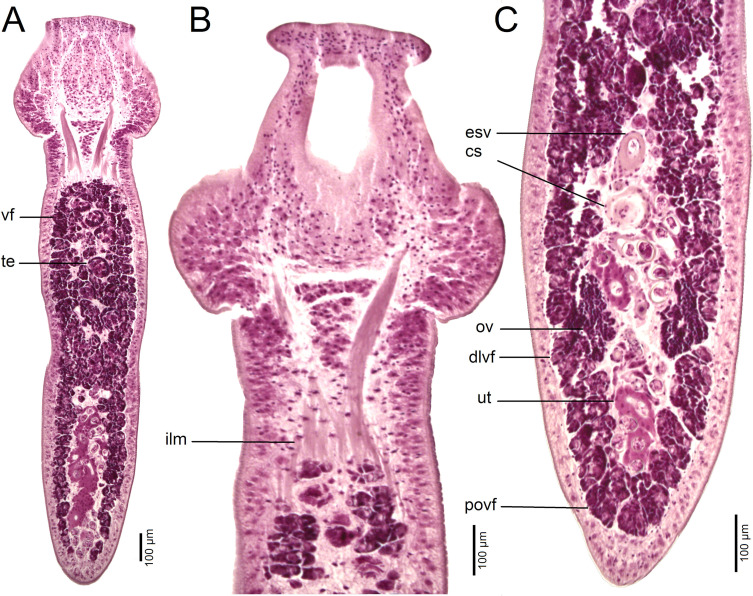
Histological section of *Archigetes megacephalus* n. sp. from *Ictiobus cyprinellus* (Valenciennes) (US 242a, IPCAS C-904/1, PBI-462), Chotard Lake near Vicksburg, Mississippi, USA. A – longitudinal section of body (total view); B – longitudinal section of scolex; D – longitudinal section of posterior part of body. Abbreviations: dlvf – dorsolateral vitelline follicles, cs – cirrus-sac, esv – external seminal vesicle, ilm – inner longitudinal muscles, ov – ovary, povf – postovarian vitelline follicles, te *–* testes, ut – uterus, vf *–* vitelline follicles.

*Description* (based on whole mounts of 16 specimens; for measurements – see Table 2): Caryophyllidea, Capingentidae *sensu* Scholz et al. [50]). Body elongate, with maximum width near mid-length of body or at level of cirrus-sac, slightly and gradually tapering towards neck region anteriorly, with posterior part of body tapering from ovarian level (Figs. 6A, 7A, 8A). Body covered with acicular fillitriches. Scolex bothrioloculodiscate (see Fig. 5.4 in Mackiewicz [30]), spherical, robust, conspicuously (at least two times) wider than neck and body, with one pair of deep, ovoid acetabulum-like median loculi, two pairs of slightly shallower, but distinct lateral loculi, and widely convex apical disc (Figs. 6A, 6B; 7A–7D; 8A, 8B). Neck distinct, narrow, relatively short. Internal and external longitudinal muscles well-developed. Osmoregulatory canals narrow, numerous, in cortex.

Testes medullary, subspherical to widely oval (Figs. 6B, 6C). Anterior-most testes begin posterior to anterior-most vitelline follicles. Posteriorly, testes reach to level of external seminal vesicle (Fig. 6C), rarely to cirrus-sac level. Cirrus-sac spherical, thick-walled, anterior to ovary. External seminal vesicle elongate, thick-walled (Fig. 6C). Male genital pore anterior to female gonopore, opens separately to common genital atrium.

Ovary butterfly-shaped, follicular (Figs. 6C, 8C). Vagina tubular, sinuous, widened to form elongate, narrow seminal receptacle anterodorsal to ovarian isthmus, joins uterus to form uterovaginal canal, opening close to but separate from male gonopore at bottom of distinct genital atrium (see Fig. 5.24 of Mackiewicz [30]) (Fig. 6C).

Preovarian vitelline follicles numerous, in medullary parenchyma (Figs. 6B, 6C; 8A), almost always connected with postovarian group of vitelline follicles by continuous row of follicles situated alongside, laterodorsal to ovarian wings (Figs. 6C; 8A, 8C). Postovarian vitelline follicles relatively numerous, forming V-shaped field (Fig. 6C; Table 2).

Uterus forms several loops, one loop extending slightly anterior to cirrus-sac (Fig. 6C, 8C); uterine glands well-developed, absent only in distal and proximal parts of uterus. Eggs operculate, without fully formed oncosphere *in utero*.

#### Differential diagnosis

The new species differs from other congeneric species, including *Archigetes loculotruncatus* n. sp., by the shape and relative size of the scolex, which is bothrioloculodiscate and conspicuously (> 2×) wider than the body (Figs. 6A, 6B; 7A–7D; 8A, 8B). The new species can also be distinguished from *A. loculotruncatus* n. sp. by the presence of vitelline follicles nearly always present alongside the ovarian wings, whereas only some specimens of *A. loculotruncatus* n. sp. have a few follicles laterodorsal to the ovary and they never form a continuous row as in *A. megacephalus* n. sp. (compare Figs. 2C, 3C and 6C). In addition, the new species can be distinguished by some subtle biometrical differences (see also Table 2).

#### Remarks

*Archigetes megacephalus* n. sp. was found in all three species of buffalo (*Ictiobus* spp.) examined from Chotard Lake (western part of Mississippi State), and in smallmouth buffalo from Reelfoot Lake, Tennessee. The new species was found sympatric with *A. loculotruncatus* n. sp. in the anterior part of the intestine in three fish individuals, one black buffalo (US 244) and two smallmouth buffalo (US 257; RF3/490). These new species can be distinguished from each other mainly by the shape and size of the scolex and body length, but live cestodes are highly mobile and change shape, including the scolex. For this reason, identification of live specimens with the naked eye can be difficult in mixed infections. Specimens should be fixed with hot fixative so that they retain their natural shape and do not shrink unnaturally, as is typical for specimens fixed with unheated fixative.

#### *Archigetes vadosus* n. sp. (Figs. 9, 10; Table 2)

urn:lsid:zoobank.org:act:21724C2F-6202-4E5D-A514-D9D832598A12

**Figure 9:**
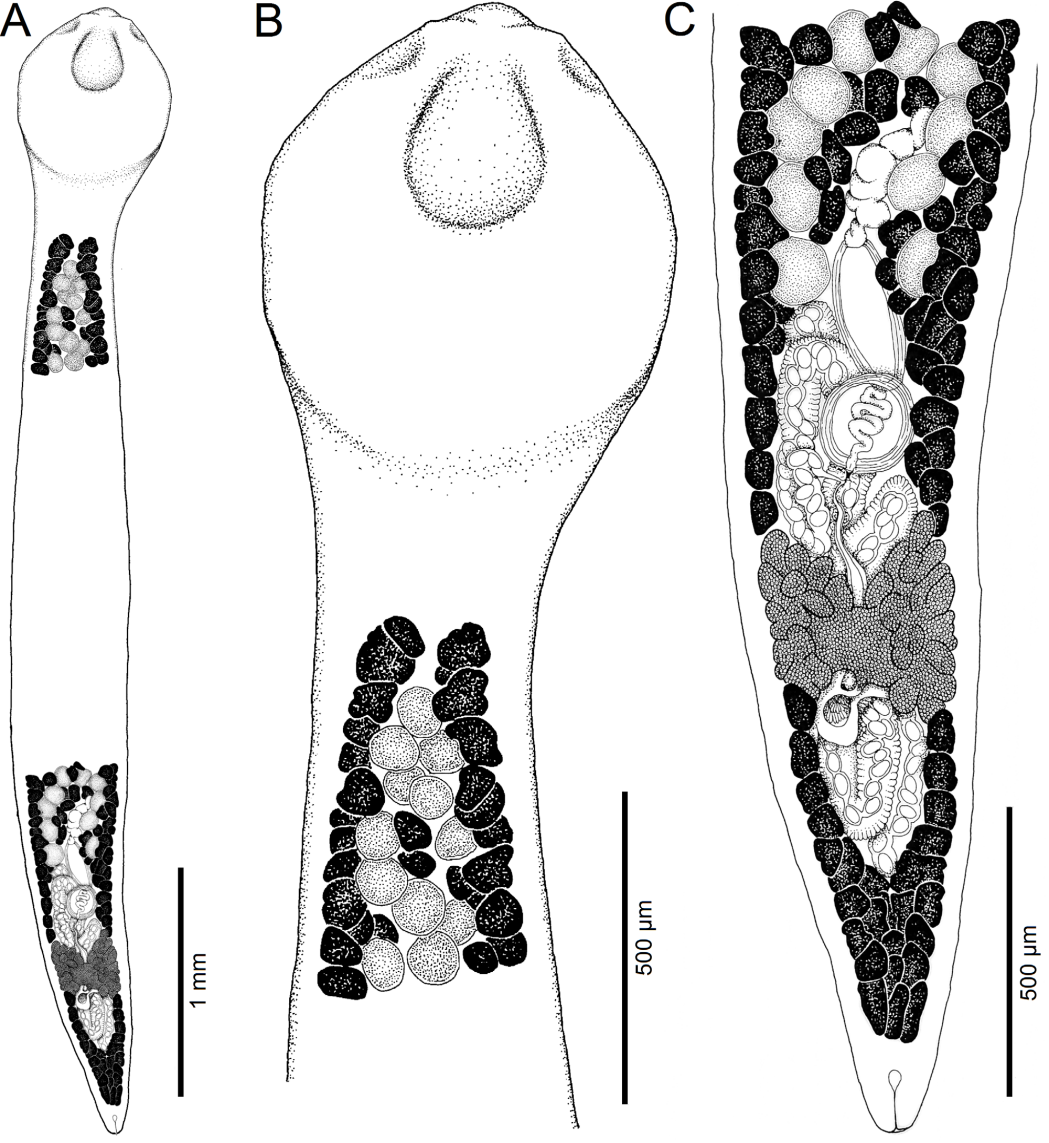
Line drawings of *Archigetes vadosus* n. sp. from *Ictiobus bubalus* (Rafinesque), Bluff Creek, Pascagoula River, Mississippi, USA (host field codes US 862d, IPCAS C-905/1). A *–* total view (testes and vitelline follicles are not illustrated in the middle portion of the body), B *–* anterior part with scolex, C *–* posterior part.

**Figure 10:**
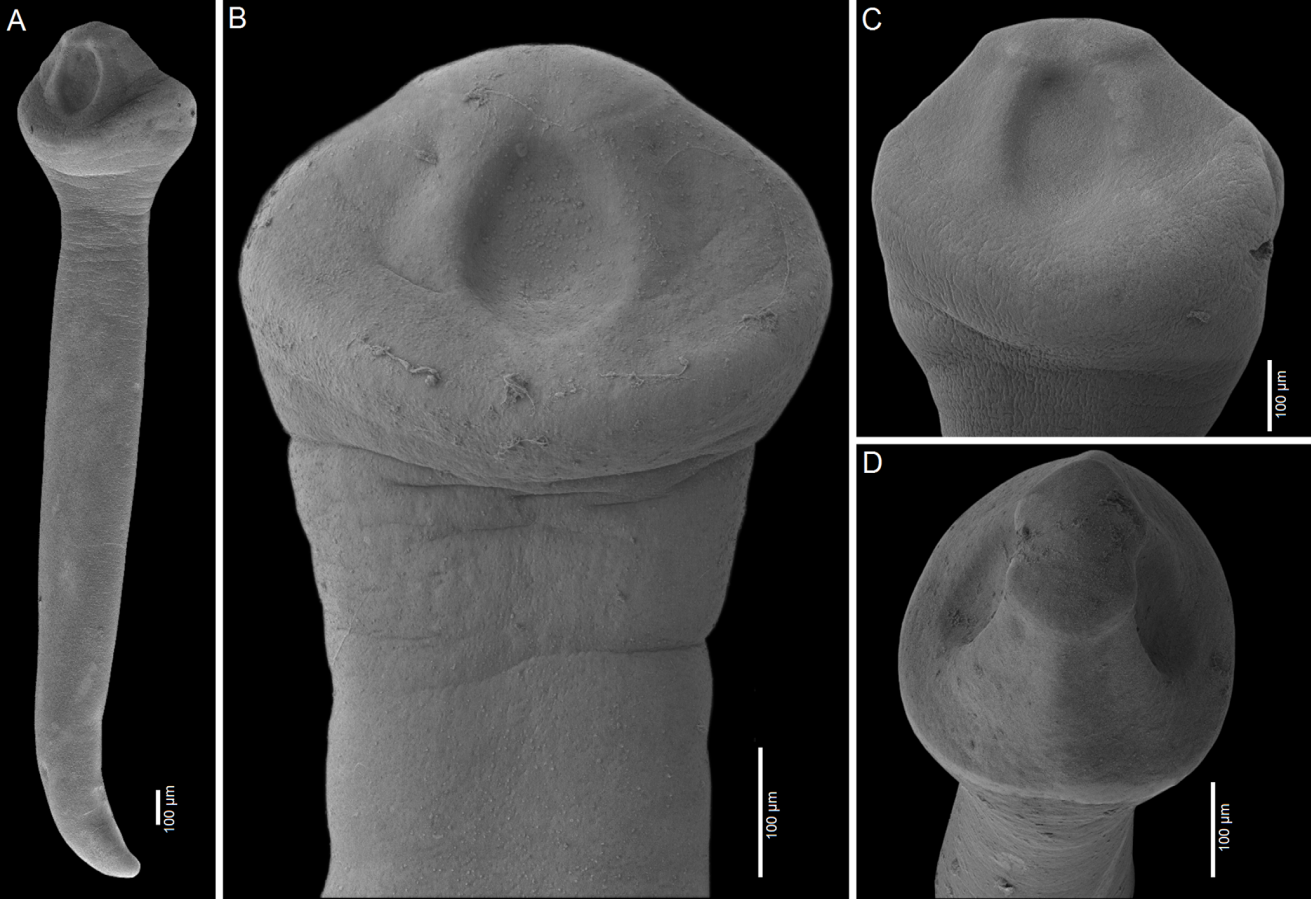
Scanning electron micrographs of *Archigetes vadosus* n. sp. from *Ictiobus bubalus* (Rafinesque) A, C – specimens from host field codes US 831d, Oxbow south of Cumbest Bridge landing, Pascagoula River, Mississippi, USA; B – specimen from host code FR19_765, Sunflower River in Indianola, Mississippi, USA; D – specimen from host code US 862d (all specimens IPCAS C-905/1), Bluff Creek, Pascagoula River, Mississippi, USA. A – total view, B – detail of scolex, C, D – variability in scolex shape.

*Type host*: *Ictiobus bubalus* (Rafinesque), Smallmouth buffalo (Cypriniformes: Catostomidae, Ictiobinae).

*Site in host*: Anterior intestine.

*Type locality*: Bluff Creek, Mississippi, USA (30.4568653; −88.6180754).

*Additional localities*: Pascagoula River and Sunflower River, Indianola (all Mississippi, USA), Reelfoot Lake (Tennessee, USA).

*Type material*: Holotype from *I. bubalus* (host code No. US 862d) collected on 23 June 2019 (IPCAS C-905/1); two paratypes (hologenophores) from *I. bubalus* (US 862b, c) (IPCAS C-905/1); one paratype from *I. bubalus* (US 831d-3), one paratype from *I. bubalus* (US 863c) (USNM 1661736); two paratypes from *I. bubalus* (US 869b) (HWML 216783; 216784; IPCAS C-905/1); one paratype from *I. bubalus* (US 871a) (USNM 1661737).

*Material studied*: Fifteen slides with 16 whole-mounted specimens from *I. bubalus* (US 827c, 831c, d, 862b–d, 863c, 866b, 869b and 871a) from Oxbow south of Cumbest Bridge landing on the Pascagoula River and Bluff Creek, collected by the present authors (R.K., M.O. and T.S.) on 19 and 23 June 2019; one slide with two whole-mounted specimens from *I. bubalus* (FR19_655), Oxbow on the Pascagoula River, Vancleave, coll. by F. Reyda on 12 August 2019; two slides with two whole-mounted specimens (one slide together with *A. iowensis* – see below) from *I. bubalus* (FR19_767) from Sunflower River, coll. by F. Reyda on 17 August 2019; four slides with eight whole-mounted specimens from *I. bubalus* (host code No. RF3/490), Reelfoot Lake, donated by J.S. Mackiewicz to T.S.

*Representative DNA sequences and phylogenetic relationships*: The *lsr*DNA sequences of six individuals from *I. bubalus* (US 828a, 3× 831d, 862b and 862c – OM103266 – OM103271). Sequence divergence among individual isolates of *A*. *vadosus* n. sp. was 0–4 nt (0–0.30%). They differed from sequence of *A*. *loculotruncatus* n. sp. by 17–20 nt (1.33–1.41%), from sequences of *A*. *megacephalus* n. sp. by 4–5 nt (0.30–0.37%), and from sequences of *A*. *sieboldi* by 1–5 nt (0.08–0.37%).

*Etymology*: The species name *vadosus* (= shallow in Latin) refers to the unusually shallow pair of median loculi.

*Description* (based on whole mounts of 16 specimens; for measurements – see Table 2): Caryophyllidea, Capingentidae *sensu* Scholz et al. [50]). Body elongate, with maximum width at middle of body, slightly tapering towards neck region anteriorly and narrowing gradually towards posterior end (Figs. 9A, 10A). Body covered with acicular fillitriches. Scolex bulboloculate, i.e., bulbous, wider than neck, with three pairs of loculi, one pair of shallow median ovoid loculi, two pairs of indistinct lateral loculi and indistinct apical disc forming small apical cone (Figs. 9A, 9B; 10B–10D). Neck short. Internal and external longitudinal muscles well-developed. Osmoregulatory canals numerous, narrow, in cortex.

Testes medullary, subspherical to widely oval (Fig. 9B, 9C). Anterior-most testes begin posterior to anterior-most vitelline follicles. Posteriorly, testes reach to level of external seminal vesicle or to anterior margin of cirrus-sac (Fig. 9C). Cirrus-sac subspherical to spherical, thick-walled. External seminal vesicle elongate, thick-walled. Male genital pore opens anterior to female gonopore to common genital atrium.

Ovary butterfly-shaped, follicular (Fig. 9C). Vagina tubular, sinuous, widened to form elongate, narrow seminal receptacle anterodorsal to ovarian isthmus, joins with uterus to form uterovaginal canal, opening close to but separate from male gonopore (Fig. 9C) at bottom of distinct genital atrium (see Fig. 5.24 of Mackiewicz [30]). Preovarian vitelline follicles numerous, in medullary parenchyma, reaching posteriorly to ovary, but always absent alongside ovary (Fig. 9C). Postovarian vitelline follicles relatively numerous, reaching ovary, forming V-shaped field (Fig. 9C).

Uterus forms several loops, one loop extending anterior to cirrus-sac (Fig. 9C); uterine glands well-developed, absent only in distal and proximal parts of uterus. Eggs operculate, without fully formed oncosphere *in utero*.

#### Differential diagnosis

The new species differs from the five previously known species of *Archigetes* by having an elongate body with a distinct neck, and from the two new species by possessing a bulboloculate scolex with shallow median loculi, almost indistinct lateral loculi and indistinct apical disc (Figs. 9A, 9B; 10B–10D). *Archigetes vadosus* n. sp. can also be distinguished from most congeners by the complete absence of vitelline follicles alongside the ovarian wings (present in other species). Other biometrical differences from *A. loculotruncatus* n. sp. and *A. megacephalus* n. sp. are obvious in Table 2. The new species is closely related to *A. sieboldi* (Fig. 1), but differs considerably by body shape, especially the presence of a distinct neck region which separates the scolex from the remaining body, a butterfly-, rather than dumb-bell-shaped ovary, and the external seminal vesicle longer than the diameter of the cirrus-sac.

#### Remarks

*Archigetes vadosus* n. sp. was found in only one species of buffalo, *I. bubalus*, in the Pascagoula River, neighbouring Bluff Creek, Mississippi, and Reelfoot Lake, Tennessee. One smallmouth buffalo from Reelfoot Lake harboured all three new species, one *A. loculotruncatus*, five *A. megacephalus*, and eight *A. vadosus*. Interestingly, *A. vadosus* n. sp. was not found in Chotard Lake, where two other new species commonly occurred in two smallmouth buffaloes examined.

#### *Paraglaridacris limnodrili* (Yamaguti, 1934) Mackiewicz, 1994 (Fig. 11)

Syns.: *Glaridacris limnodrili* Yamaguti, 1934; *Archigetes limnodrili* (Yamaguti, 1934) Kennedy, 1965; *Archigetes iowensis* Calentine, 1962 (new synonym)

**Figure 11:**
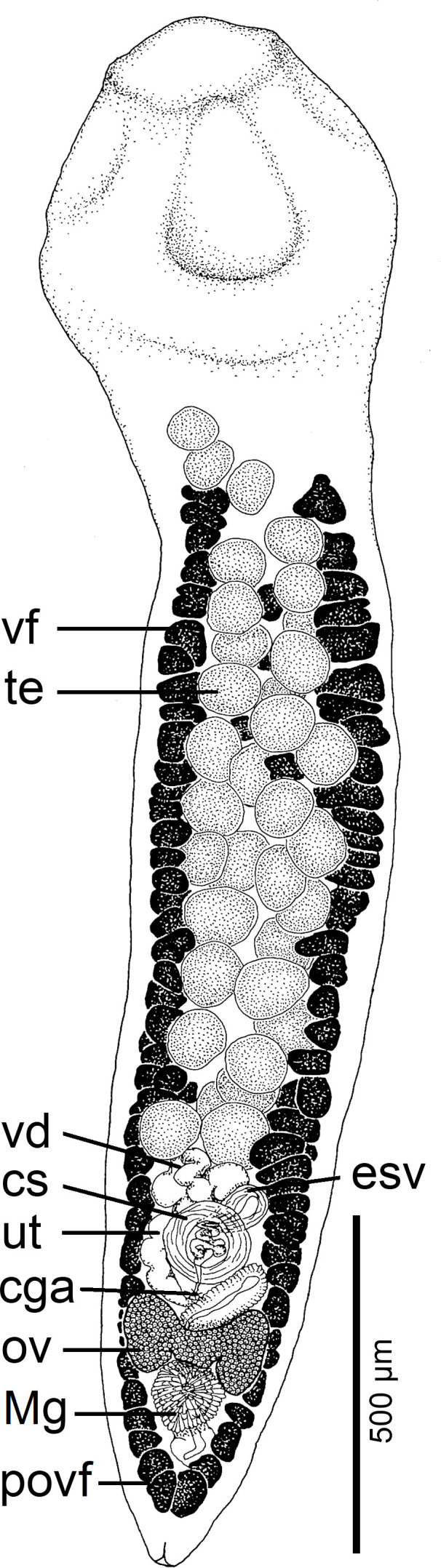
Line drawing of *Paraglaridacris limnodrili* (Yamaguti, 1934) Mackiewicz, 1994 (synonym *Archigetes iowensis* Calentine, 1962). from *Ictiobus bubalus* (Rafinesque) (FR19_767, IPCAS C-588/3), Sunflower River in Indianola, Mississippi, USA. Abbreviations: cga – common genital atrium, cs – cirrus-sac, esv – external seminal vesicle, Mg – Mehlis’ gland, ov – ovary, povf – postovarian vitelline follicles, te *–* testes, ut – uterus, vd *–* vas deferens, vf *–* vitelline follicles.

*Type host*: *Cyprinus carpio* Linnaeus, Common carp (Cypriniformes: Cyprinidae).

*Other hosts*: *Ictiobus bubalus* (Rafinesque), Smallmouth buffalo (Cypriniformes: Catostomidae, Ictiobinae) (new host record); *Limnodrilus hoffmeisteri* Claparède, Red worm (Clitellata: Naididae) (progenetic plerocercoids).

*Site in host*: Anterior intestine (fish) and body cavity (naidids).

*Type locality*: Iowa River, Hardin County, Iowa, USA.

*Distribution*: Whole USA (Iowa, Mississippi – new geographical record, Wisconsin).

*Type specimens*: Holotype from *C. carpio* (USNM 1355440); paratype from *L. hoffmeisteri* (USNM 1355441).

*Material studied*: One specimen from the intestine of *I. bubalus* (host code No. FR-19_767; IPCAS C-588/3), Sunflower River, Indianola, Mississippi, collected by F. Reyda on 17 August 2019; one specimen from the body cavity of *L. hoffmeisteri*, locality unspecified, donated by J.S. Mackiewicz to T.S. (IPCAS C-588/1); four whole-mounted specimens and two slides with longitudinal sections of another specimen from *Cyprinus carpio* (BN C-78), Wisconsin, USA, coll. by R.L. Calentine in 1966 and donated by J.S. Mackiewicz to T.S. (IPCAS C-588/2).

*Representative DNA sequences*: Not available.

*Redescription* (based on six specimens; for measurements – see Table 3): Caryophyllidea, Capingentidae *sensu* Scholz et al. [50]). Body narrowly ovoid, with maximum width at level of cirrus-sac or external seminal vesicle, slightly tapering towards neck region (Fig. 11). Scolex bothrioloculodiscate (see Fig. 5.4 in Mackiewicz [30]), slightly wider than neck, with one pair of shallow acetabulum-like loculi, two pairs of indistinct lateral loculi, and well-developed apical disc (Fig. 11). Neck short, but distinct, slightly narrower than body width. Internal and external longitudinal muscles well-developed. Osmoregulatory canals narrow, numerous, in cortex.

**Table 3:** Comparative measurements of *Paraglaridacris limnodrili* (Yamaguti, 1934) Mackiewicz, 1994 (synonym *Archigetes iowensis* Calentine, 1962).

Host	*Pseudogobio esocinus* (type host)^1^	*Cyprinus carpio* (type host)	*Cyprinus carpio*	*I. bubalus*	*Limnodrilus hoffmeisteri*		
	*Misgurnus fossilis* 1 ^,^ 2	*Limnodrilus hoffmeisteri*					
	*Limnodrilus* sp.^1^^,^ 3						
State/country	JP^1^, UA^2^, GB^3^	IA, USA	WI, USA	MS, USA	unknown, USA		
	Protasova et al [43]	Calentine [5]					
Character/no. of specimens		*n* = 20	*n* = 4	*n* = 1	*n* = 1		
Body length (mm)	1.5–2.5^1^	1.1–2.4	2.6–3.7	2.3	2.2		
	2.5^2^						
	1.1–2.1^3^						
Maximum width	0.3–0.8^1^	370–720	451–667	397	457		
	0.4–0.5^2^						
	0.3–0.5^3^						
Width at cirrus-sac level	–	–	427–654	320	409		
Scolex length	–	–	458–587	504	337		
Width	–	–	468–598	536	495		
Neck length	–	–	–	75	–		
width	–	–	342–514	386	416		
Testis size (length × width) number	–	190 × 40 (mean)	78–113 × 65–102 (*n* = 12)	90–113 × 84–97 (*n* = 10)	73–85 × 53–64 (*n* = 10)		
	40^1^	57–76	54–89	35	59		
	48–50^2^						
	39–63^3^						
Distance from first vitelline follicles	–	–	89–129	66	97		
Distance from anterior extremity	–	–	435–584	623	192		
Testicular area length (mm)	–	–	1081–1520	1063	876		
Cirrus-sac size (length × width)	120 × 110^2^	140–170 (in diameter)	146–162 × 150–178	126 × 133	158 × 162		
Ovary width	280^1^	–	332–433	214	291		
Ovary structure	–	Compact	Compact	Compact	Compact		
Ovary shape	–	Dumbbell	Dumbbell	Dumbbell	Dumbbell		
Length of ovarian wings	170^1^	90–230	150–248	137	206		
Width of ovarian wings	–	–	103–186	75	116		
Vitelline follicle size (length × width)	–	80 × 30 (mean)	54–107 × 51–89 (*n* = 12)	50–67 × 33–52 (*n* = 10)	70–87 × 31–55 (*n* = 10)		
Distance of anterior-most vitelline follicles from Anterior extremity	–	–	522–705	579	285		
Length of uterine area	–	–	533–698	485	589		
Size of intrauterine eggs	50–57 × 30–36^1^	43 × 32 (mean)	44–52 × 30–38 (*n* = 16)	No present	43–50 × 27–36 (*n* = 10)		
	51–58 × 32–34^2^						
	45–63 × 27–36^3^						
External seminal vesicle (length × width)	–	90–120 × 70–100	118–124 × 88–104	81 × 63	150 × 91		
Distance of genital atrium from posterior end	–	–	698–885	439	822		
Postovarian vitelline follicle field shape	–	Present	U	U	U		

1 Yamaguti [61].

2 Kulakovskaya [22].

3 Kennedy [19]

Testes medullary, subspherical to widely oval (Fig. 11), with first testes anterior to anterior-most vitelline follicles. Posteriorly, testes reach to cirrus-sac (Fig. 11). Cirrus-sac subspherical, thick-walled. External seminal vesicle ovoid, thick-walled, shorter than cirrus-sac. Male genital pore anterior to female gonopore, opens to common genital atrium.

Ovary compact (non-follicular), dumb-bell-shaped (Fig. 11). Vagina tubular, slightly sinuous, widened to form elongate, narrow seminal receptacle anterodorsal to ovarian isthmus, joins uterus to form uterovaginal canal that opens separately from male gonopore into small, but distinct genital atrium (corresponding to Fig. 5.24 of Mackiewicz [30]; Fig. 11). Preovarian vitelline follicles numerous, in medullary parenchyma. Postovarian vitelline follicles relatively numerous, forming U-shaped field (Fig. 11; Table 3). Fields of preovarian and postovarian vitelline follicles continuous, connected by follicles dorsolateral to lateral ovarian wings.

Uterus forms several loops, with distal part of uterus not extending farther anteriorly than cirrus-sac (Fig. 11). Uterine glands well-developed, absent only in distal and proximal parts of uterus. Eggs operculate, without fully formed oncospheres *in utero*.

#### Remarks

Calentine [5] described *A. iowensis* based on adults found in common carp and progenetic plerocercoids in the naidid *L. hoffmeisteri* from the Iowa River. The same author [6] studied the life cycle of this species in laboratory conditions. Thereafter, the species was found in common carp from the Red Cedar River in Wisconsin by D.R. Sutherland in 1979 (voucher deposited as USNM 1397329), but this record does not seem to have been published. Williams [58] also found *A. iowensis* in *L. hoffmeisteri* in Wisconsin (USNM 1370351, 1370352), but designated his specimens as “*Archigetes limnocesti*”, which is a non-existing name (*nomen nudum*).

Comparison of North American specimens of *A. iowensis* from cypriniforms and oligochaete host with those of *Paraglaridacris limnodrili* from the Palearctic region has revealed that these species can be distinguished from each other neither by their morphology nor measurements [18, 43, 61].

Both species are typified by (i) a compact, dumb-bell-shaped ovary; (ii) a small body separated by a short, but distinct neck, which is slightly narrower than the scolex, anterior-most testes being always anterior to anterior-most vitelline follicles; (iii) largely lateral preovarian vitelline follicles, with a very few median folllicles (Calentine [5] originally the preovarian vitelline follicles were reported as exclusively lateral, but Mackiewicz [30] corrected this characteristic); (iv) vitelline follicles uninterrupted alongside (dorsolateral) the ovarian wings, thus making bands of pre- and postovarian follicles continuous; (v) the uterus reaching anteriorly only to the anterior margin of the cirrus-sac; and (vi) external seminal vesicle shorter than the diameter of the cirrus-sac. *Archigetes iowensis* and *P. limnodrili* have been found as adults in cypriniforms and as progenetic plerocercoids in oligochaetes [5, 61]. Based on the rule of priority, *A. iowensis* becomes a junior synonym of *P. limnodrili* and the number of valid species of *Archigetes* is reduced to six (three new species, *A. sieboldi*, *A. brachyurus* and *A. cryptobothrius*).

#### *Archigetes sieboldi* Leuckart, 1878

Syns.: *Archigetes appendiculatus* Mrázek, 1898; *Biacetabulum sieboldi* Szidat, 1937; *Archigetes* sp. 3 of Scholz and Pérez-Ponce de León [49] (new synonym)

*Type host*: *Limnodrilus hoffmeisteri* Claparède, Red worm (Clitellata: Naididae).

*Other hosts* (in North America only; all hosts see in Protasova et al. [43]): Common carp, *Cyprinus carpio* (Cypriniformes: Cyprinidae); shortfin silverside, *Chirostoma humboldtianum* (Valenciennes) (Atheriniformes: Atherinidae) (new host record).

*Site in host*: Body cavity (progenetic plerocercoids in oligochaetes); intestine (adults in fish).

*Type locality*: Ponds and puddles around Leipzig, Germany.

*Distribution*: Kinnickinnic River, River Falls, Wisconsin, USA [4]; Lago de Zacapu, Michoacán, Mexico [49] (for record out of North America see in Protasova et al. [43]).

*Morphological description*: Mrázek [32], Kennedy [19], Calentine and DeLong [4], Protasova et al. [43].

*Material studied*: seven plerocercoids from *L. hoffmeisteri* and *L. udekemianus* Claparède; two adults from *C. carpio*, both from several fishponds in South Bohemia, Czech Republic, collected by F. Moravec and T.S. between 1984 and 1988 (IPCAS C-45/1, C-45/5, and C-45/2, respectively) [31]; 13 adults from *C. carpio*, Tisa River near Velké Trakany, Slovakia, coll. by M.O. and V. Hanzelová in June 2006 and 2007, and April 2008 (host code Nos. 263/06, 155/07, 159/07, 17/08 and 20/8); one adult from *Blicca bjoerkna* (L.), Latorica River, Slovakia, coll. by R. Ergens in 1964 (IPCAS C-45/3); two adults from *Abramis brama* (L.), two fishponds in Průhonice park near Prague and České Budějovice, respectively, coll. by T.S. in 1983 and 1990 (IPCAS C-45/4); two adults from *Pseudorasbora parva* (Temminck & Schlegel), and two adults from *Rhynchocypris lagowskii* (Dybowski), Lake Suwa, Japan, coll. by T. Shimazu in 1992 (IPCAS C-45/6 and C-45/8, respectively); six adults from *Gnathopogon elongatus* (Temminck & Schlegel), Kawashima, Japan, coll. by M. Urabe in 2002, 2003 and 2012 (IPCAS C-45/7); six plerocercoids from *Limnodrilus udekemianus*, Latka River near Borok, Yaroslavl Region, Russia, coll. by L.G. Poddubnaya in 2000 (IPCAS C-368/1); one specimen from *Chirostoma humboldtianum,* Lago de Zacapu, Michoacán, collected by Berenit Mendoza-Garfias in November 2008 (CNHE 6801; identified as *Archigetes* sp. 3 by Scholz and Pérez-Ponce de León [49]).

*Representative DNA sequences and phylogenetic relationships*: Olson et al. [36] sequenced adult *A. sieboldi* from the fish host *Gnathopogon elongatus* (Temminck & Schlegel, 1846) in Japan and progenetic plerocercoid from the coelom of oligochaete *L. hoffmeisteri* in Russia: EU343744, EU343745 (*ssr* DNA; both sequences identical) and EU343736 (*lsr*DNA). Scholz et al. [50] sequenced adult *A. sieboldi* from gudgeon, *Gobio gobio* L. (Cyprinidae) from the Czech Republic: MW027431 (*ssr* DNA) and MW027492 (*lsr* DNA). The *lsr* DNA sequences of this species from both studies differed by 3 nt (0.24%).

#### Remarks

Calentine and DeLong [4] and Calentine [7] studied the life history of *A. sieboldi* from the Kinnickinnic River in Wisconsin. The authors concluded that gravid stages occur in oligochaetes and occasionally in fish because a high prevalence of infection with *A. sieboldi* in oligochaetes compared to its rare occurrence in fish indicates that *A. sieboldi* is primarily a parasite of oligochaetes. Wiśniewski [60] and Nybelin [34] in Europe failed to infect cyprinids, but Kulakovskaya [23] successfully infected cyprinids (tench – *Tinca tinca* (L.)) with *A. sieboldi* from oligochaetes.

The specimen from *Chirostoma humboldtianum* in Mexico, designated as *Archigetes* sp. 3 by Scholz and Pérez-Ponce de León [49] (CNHE 6801), corresponds in its morphology to *A. sieboldi* found by Calentine and DeLong [4] in the USA, including overall shape of the body, with the scolex of a similar width as the remaining body, a more anterior position of vitelline follicles compared to that of the anterior-most testes, follicular, dumb-bell-shaped ovary, preovarian vitelline follicles lateral and median, with follicles lacking at the ovarian level, thus making pre- and postovarian vitelline follicles separated. Therefore, this specimen is considered to belong to *A. sieboldi* from *C. humboldtianum* in Mexico and represents a new definitive host and new geographical area, but this fish species should be confirmed as a true host of *A. sieboldi*.

#### Unidentified species of *Archigetes*

Scholz and Pérez-Ponce de León [49] reported three morphotypes of *Archigetes* from two species of shiners, *Notropis* spp., and *Chirostoma humboldtianum* in the Nearctic part of Mexico. These specimens differ conspicuously from the newly described species from the USA in their much smaller size (total length up to 2.50 mm), dumb-bell-shaped ovary, uterine loops reaching far anterior to the cirrus-sac, and the external seminal vesicle smaller than the cirrus-sac.

All the three morphotypes can be distinguished from *Paraglaridacris limnodrili* (syn. *A. iowensis*) by the lateral and median position of preovarian vitelline follicles (follicles form two lateral bands only, with only very few median follicles in *A. iowensis*), and by much shallower loculi on the scolex in Mexican specimens (conspicuous, deep loculi in *A. iowensis*). The third morphotype from *C. humboldtianum* (*Archigetes* sp. 3) resembles in its morphology *A. sieboldi* found by Calentine and DeLong [4] in North America and is considered conspecific (see above).

Camp [8] found two caryophyllideans identified as *Archigetes* sp. in creek chub, *Semotilus atromaculatus* Mitchill (Cypriniformes: Leuciscidae), Sugar Creek, Normal, Illinois, USA. Voucher specimens were not available to the present authors.

### Key to the identification of species of *Archigetes* Leuckart, 1878 and *Paraglaridacris* Janiszewska, 1950 in North America

Because of the lack of newly collected or museum material of *A. brachyurus* and *A*. *cryptobothrius*, the identification key includes only five species of both morphologically similar genera that occur in North America. Morphotypes *Archigetes* sp. 1 and sp. 2 from Mexico are not included (see above).

1a. Ovary compact; anterior-most testes anterior to anterior-most vitelline follicles; median vitelline follicles almost completely absent (a few follicles may be present medially) ........................................................................................................................... *P. limnodrili* (synonym *A. iowensis*)
1b. Ovary follicular; anterior-most testes posterior to anterior-most vitelline follicles; median vitelline follicles present ...... 2
2a. Ovary butterfly-shaped; neck distinct, narrower than the body .............................................................................. 3
2b. Ovary dumb-bell-shaped; neck indistinct, wider than the scolex ............................................................ ***A*. *sieboldi***
3a. Body short (length < 5 mm), scolex bothrioloculodiscate or bulboloculate .............................................................. 4
3b. Body long (length > 5 mm), scolex loculotruncate ........................................................... ***A. loculotruncatus* n. sp.**
4a. Scolex bothrioloculodiscate, conspicuously (> 2×) wider than the body .............................. ***A. megacephalus* n. sp.**
4b. Scolex bulboloculate, only slightly wider than the body ............................................................... ***A. vadosus* n. sp.**

## Discussion

Previously, only two nominal taxa of *Archigetes* have been reported in North America [4–7, 58]. New data greatly expand the known spectrum of definitive hosts for *Archigetes* species that now include also catostomids (suckers). Three new species were found in the southern part of the USA, whereas *Paraglaridacris limnodrili* (new syn. *A. iowensis*) and *A. sieboldi* were previously known only from the Midwest (Iowa and Wisconsin). The present study, together with previous studies by the present authors, clearly demonstrates that the Nearctic fauna of freshwater fish tapeworms is still severely underexplored and insufficiently known [21, 47].

All new North American species of *Archigetes* have an elongate body with a distinct, relatively long neck (usually absent or indistinct in species described in Europe), butterfly-shaped ovary, and a conical to globular scolex wider than the neck. The new species also share the presence of a small, shallow genital atrium to which the male and female (uterovaginal canal) gonopores open separately, thus not forming a single hermaphroditic duct. The presence of a single common gonopore or two separate gonopores has been considered taxonomically important at the genus-level. Mackiewicz [30] distinguished four types, but some of them are difficult to differentiate from each other, depending in part on the contraction or relaxation of the worm.

Species of *Archigetes* and the closely related genus *Biacetabulum* Hunter, 1927 should have a single gonopore according to Mackiewicz [30]. However, recent studies of *Biacetabulum* spp. have shown that the male and female gonopores are separate and do not form a hermaphroditic duct, but open into a shallow genital atrium (see [54, 55]). Thus, using the terminology of Mackiewicz [30] and his key to the genera of the Caryophyllidea, all species of these two genera would be classified as having separate gonopores, but in fact have a single pore – a mouth of the common genital atrium. This situation is consistent with observations of Yamaguti [61] and Protasova et al. [43].

Johnston and Muirhead [17] described, based on a single specimen, *Biacetabulum tandani* Johnston and Muirhead, 1950 from the plotosid catfish *Tandanus tandanus* (Mitchell) in Australia. The authors placed the species in *Biacetabulum* because of the anterior extent of the uterus far anterior to the cirrus-sac and the presence of two acetabulum-like loculi on the scolex. Scholz and Oros [48] recognised this species as valid, but Uhrovič et al. [56] questioned its generic assignment. However, *B. tandani* is provisionally retained in *Biacetabulum* until new material is available. The most intriguing features of this poorly known species are its occurrence in a very distant zoogeographical distribution from that of species of both genera, which occur in the Palaearctic and Nearctic zoogeographical regions, and the presence of an external seminal vesicle, which is a morphological characteristic present only in most species of the Capingentidae, including *Archigetes* and *Biacetabulum* (see [50]). A feasible explanation of the occurrence of *B. tandani* in Australia could be possible introduction of this species with imported common carp to Australia and subsequent host switch to local plotosid catfish which may represent an accidental host. However, there is no supporting evidence for this assumption because no data are available on the occurrence of caryophyllidean tapeworms in introduced common carp in Australia.

One of the interesting results of molecular analysis is that both European isolates of *A. sieboldi* fall within the same clade as *A. vadosus* n. sp. However, these tapeworms are not considered conspecific because they differ considerably from each other in their morphology, including shape of the ovary (butterfly-shaped in the new species versus dumb-bell-shaped in *A. sieboldi*), different spectrum of fish hosts (Catostomidae versus Cyprinidae) and partly zoogeographical distribution (southern USA versus Holarctic, with most records in Europe). Comparison of sequence data from *A. sieboldi* from North America with *A. vadosus* could be quite instructive, but no ethanol-fixed material of *A. sieboldi* from North America is available. Another obstacle to better understand relationships between caryophyllidean tapeworms is the existence of multiple mt haplotypes that most likely represent paralogs (i.e. numts), which were detected by Brabec et al. [3] in two mitochondrial genes (*cox*1 and *nad*3).

Molecular phylogenetic analysis also revealed that four North American species of *Archigetes* form a monophyletic group nested within the clade composed of *Biacetabulum* spp. (Fig. 1), which is consistent with the results in Scholz et al. [50]. The species of *Archigetes* form a sister lineage to the clade composed of the three so-called long-necked, recently described species of *Biacetabulum* from redhorses and spotted suckers [54].

The close relationship between the species of *Archigetes* and *Biacetabulum* is not surprising, and some species have been placed in both genera, such as *Caryophyllaeus appendiculatus* Ratzel, 1868 (probably larva of *Caryophyllaeus laticeps* (Pallas, 1781)) in *Archigetes* and *Biacetabulum* [19]. Hunter [14] mentioned close morphological similarity between these genera when he erected *Biacetabulum* and differentiated them only by exclusive egg production of progenetic plerocercoids in oligochaetes for species of *Archigetes* versus full maturation exclusively in teleosts for species of *Biacetabulum*.

Based on the ICZN [15] priority rule, *Archigetes* has priority and all species of *Biacetabulum* would be transferred to this genus, provided that both genera are considered synonymous. Unfortunately, no molecular data are available for the type species of *B. infrequens* Hunter, 1927. Therefore, the synonymy of the two genera is not yet proposed. However, these two genera could be distinguished from each other by a somewhat smaller body in *Archigetes* (less than 8 mm, usually less than 5 mm in *Archigetes vs* 4–16 mm in *Biacetabulum*), an H-shaped ovary in *Biacetabulum vs* dumb-bell-shaped ovary in *Archigetes*, the extent of vitelline follicles, which may be present alongside the ovary in *Archigetes* but are absent in *Biacetabulum*, and uterine loops extending slightly anterior to the cirrus-sac in *Archigetes vs* uterine loops extending far anterior to the cirrus-sac in *Biacetabulum.* The addition of three new species with a larger body and a butterfly-shaped ovary implies that the generic diagnosis of the genus should be amended:

### *Archigetes* Leuckart, 1878

*Amended generic diagnosis:* Caryophyllidea: Capingentidae *sensu* Scholz et al. [50]. Body small (less than 10 mm, usually only a few mm), with maximum width most commonly at middle part of body. Inner longitudinal musculature well developed. Scolex bothrioloculodiscate, globulate or loculotruncate. Neck present or indistinct. Testes medullary. Cirrus-sac thick-walled, subspherical to spherical, anterior to ovary. External seminal vesicle present, thick-walled. Ovary follicular, dumb-bell- or butterfly-shaped, with short, wide lateral lobes and wide ovarian isthmus. Preovarian vitelline follicles medullary, lateral and median. Postovarian follicles present, may be continuous with preovarian follicles. Uterus forms several loops, with at least one loop extending anterior to cirrus-sac; uterine glands present. Male and female gonopores separate, not forming a hermaphroditic duct, but open into distinct common genital atrium. Eggs operculate, without fully formed oncosphere *in utero*. Parasites of cyprinids (Cyprinidae) and catostomids (Catostomidae, both Cypriniformes) in Holarctic Region. Progenetic plerocercoids in coelom of naidid oligochaetes.

**Type species**: *Archigetes sieboldi* Leuckart, 1878.

**Additional species**: *Archigetes brachyurus* Mrázek, 1908; *Archigetes cryptobothrius* Wiśniewski, 1928; *Archigetes loculotruncatus* n. sp.; *Archigetes megacephalus* n. sp.; *Archigetes vadosus* n. sp.

The taxonomy of the genus *Archigetes* is still insufficiently clear because European taxa were described superficially, without any type or voucher specimens deposited in collections. *Archigetes brachyurus* and *A. cryptobothrius* were described in the first third of the 20th century from oligochaetes *L. hoffmeisteri* from the Czech Republic and Poland, respectively [33, 59]. Both species seem to differ from each other especially in the shape of the scolex, with *A. brachyurus* having a hexagonal scolex with deep loculi and a distinct, narrow neck (see [33]), whereas *A. cryptobothrius* has a short scolex, only slightly wider than a wide, almost indistinct neck, and bears shallow median loculi (see [59]).

Kennedy [19] differentiated both species only by the number of testes, which in fact overlaps: 120–159 in several rows of 30–40 each in *A. brachyurus*, versus 154 in several rows of 18–26 each, in *A. cryptobothrius*. In addition, this differential criterion is questionable because of difficulties in reliably counting numerous testes and their aggregation in rows. Nevertheless, both species are considered valid by most authors [19, 43, 48]. Protasova et al. [43] reported several cyprinids as hosts of *A. brachyurus*, namely *Abramis brama* (L.), *Barbus barbus* (L.)*, Barbus petenyi* Heckel, *Gobio gobio*, and *Vimba vimba* (L.). However, no details on geographical origin (Dniester, Prut or Northern Doniets basins) and morphology of these specimens were provided. Protasova et al. [43] illustrated only progenetic plerocercoids from *L. hoffmeisteri* but these larvae identified as *A. brachyurus* differ from each other considerably in the shape of the body and scolex, and in the number of testes. *Archigetes cryptobothrius* has been reported only once since its original description, in *L. hoffmeisteri* from the Susaa River at Møllebro and Nymøllebro in Denmark [2].

Mackiewicz [30] proposed *Paraglaridacris* Janiszewska, 1950 as the first available name for *Brachyurus* Szidat, 1938, which was preoccupied by *Brachyurus* Fischer-Waldheim, 1813 (a genus of rodents). Szidat [53] erected *Brachyurus*, apparently unaware that this generic name was preoccupied, to accommodate *B. gobii* Szidat, 1938, which he found in *G. gobio* from small fishpond around the former Prussian town of Rossitten (now Rybachy in the Kaliningrad enclave of Russia) in Curonian Spit. Kennedy [19] synonymised this species with Yamaguti’s [61] *Glaridacris limnodrili* as *A. limnodrili*. Mackiewicz [30] did not question conspecificity of both taxa, but considered *G. limnodrili* to belong to *Brachyurus* Szidat, 1938, *nec* Fischer-Waldheim, 1813 (= *Paraglaridacris* sensu Mackiewicz, 1994). Comparison of *A. iowensis* specimens with *Paraglaridacris limnodrili* did not reveal any difference in their morphology and measurements in the present study (Table 3). As a result, both taxa are considered conspecific, with *A. iowensis* becoming a new junior synonym of *P. limnodrili*.

The second species, *Paraglaridacris silesiacus* Janiszewska, 1950, was described based on a single decomposed specimen from common bream, *A. brama*, in Poland [16]. The author even considered the new species description as provisional (“Comprenant qu’il s’agit d’une espèce, probablement assez rare, je résolus d’en donner une description provisoire.”). Morphological description of this species was not detailed and Janiszewska [16] did not recognise the presence of postovarian vitelline follicles, which was later corrected by Mackiewicz [27], who studied the holotype of *P. silesiacus*.

The validity of *Paraglaridacris* sensu Mackiewicz, 1994 could not be confirmed using molecular data, because no ethanol-fixed material was available. However, validity of the genus is supported by the following morphological characteristics, in which this genus differs from *Archigetes*: (i) compact ovary (versus follicular in *Archigetes*); (ii) largely lateral preovarian vitelline follicles, with only very few follicles median (median follicles are common in *Archigetes*); (iii) more anterior position of the first testes compared to vitelline follicles (versus the testes usually posterior or at same level as the anterior-most vitelline follicles). The difference in the structure of the ovary is conspicuous and enables easy differentiation between the specimens of both genera. Therefore, *Paraglaridacris* is provisionally considered a valid genus. It should also be mentioned here that two of the present authors contributed to the existing confusion as to nomenclature of these tapeworms [48].

The host specificity of most species of *Archigetes* and *P. limnodrili* was previously considered narrow, especially in North America. Calentine [6] examined 975 fishes representing 20 species from the same locality and only the common carp was infected, as in experimental infections. He also examined four species of oligochaetes but only *L. hoffmeisteri* was infected, with the same results of experimental infections of oligochaetes. A similarly narrow host range was reported for *A. sieboldi* in North America [4, 7], but a presumably conspecific tapeworm was found in *Chirostoma humboldtianum* from Mexico by Scholz and Pérez-Ponce de León [49]. The present record of *A. iowensis* in smallmouth buffalo, i.e., fish of a different family (Catostomidae), also challenges the above-mentioned assumption about strict host specificity of these species at the level of fish definitive host. Indeed, a broader host spectrum has been reported for *A. sieboldi* and *P. limnodrili* in the Palaearctic region ([43, 21], present study) and for two of the three new species of *Archigetes* described herein, because they occur in three species of fish hosts.

## Supplementary Material

The Supplementary material of this article is available at https://www.parasite-journal.org/10.1051/parasite/2022002/olm. *Table S1*: Nucleotide comparison of the partial 28S rDNA sequences of *Archigetes* spp. based on 1,356 long alignment. P-distance (%) is given above diagonal and the number of variable nucleotides below diagonal.
