# Supplementary material for: Archigetes Leuckart, 1878 (Cestoda, Caryophyllidea): diversity of enigmatic fish tapeworms with monoxenic life cycles
Source: Parasite. 2022 Feb 9;29:6. doi: 10.1051/parasite/2022002 (PMC8826581; doi:10.1051/parasite/2022002)
Supplement: Supplementary file 1 — Table S1: Nucleotide comparison of the partial 28S rDNA sequences of Archigetes spp. based on 1,356 long alignment. P-distance (%) is given above diagonal and the number of variable nucleotides below diagonal. [file parasite-29-6-s1.pdf]

**Supplementary Table S1. Nucleotide comparison of the partial 28S rDNA sequences of *Archigetes* spp. based on 1,356 long alignment. P-distance (%) is given above diagonal and the number of variable nucleotides below diagonal.**

| | | 1 | 2 | 3 | 4 | 5 | 6 | 7 | 8 | 9 | 10 | 11 | 12 | 13 | 14 |
|---|---|---|---|---|---|---|---|---|---|---|---|---|---|---|---|
| 1 | *Archigetes vadosus* n. sp. 138XXX | | 0% | 0.07% | 0.15% | 0.15% | 0.22% | 0.16% | 0.30% | 0.30% | 0.30% | 0.30% | 0.30% | 0.30% | 1.33% |
| 2 | *Archigetes vadosus* n. sp. 139XXX | 0 | | 0% | 0.15% | 0.15% | 0.22% | 0.08% | 0.22% | 0.30% | 0.30% | 0.30% | 0.30% | 0.30% | 1.33% |
| 3 | *Archigetes vadosus* n. sp. 152XXX | 1 | 0 | | 0.22% | 0.22% | 0.30% | 0.08% | 0.22% | 0.37% | 0.37% | 0.37% | 0.37% | 0.37% | 1.41% |
| 4 | *Archigetes vadosus* n. sp. 153XXX | 2 | 2 | 3 | | 0% | 0.22% | 0.16% | 0.30% | 0.37% | 0.37% | 0.37% | 0.37% | 0.37% | 1.33% |
| 5 | *Archigetes vadosus* n. sp. 13XXXX | 2 | 2 | 3 | 0 | | 0.22% | 0.16% | 0.30% | 0.37% | 0.37% | 0.37% | 0.37% | 0.37% | 1.33% |
| 6 | *Archigetes vadosus* n. sp. 15XXXX | 3 | 3 | 4 | 3 | 3 | | 0.32% | 0.37% | 0.37% | 0.37% | 0.37% | 0.37% | 0.37% | 1.33% |
| 7 | *Archigetes_sieboldi* EU343736 | 2 | 1 | 1 | 2 | 2 | 4 | | 0.24% | 0.40% | 0.40% | 0.40% | 0.40% | 0.40% | 1.46% |
| 8 | *Archigetes_sieboldi* MW027492 | 4 | 3 | 3 | 4 | 4 | 5 | 3 | | 0.44% | 0.44% | 0.44% | 0.44% | 0.44% | 1,48% |
| 9 | *Archigetes megacephalus* n. sp. 50XXXX | 4 | 4 | 5 | 5 | 5 | 5 | 5 | 6 | | 0% | 0% | 0% | 0% | 1.18% |
| 10 | *Archigetes megacephalus* n. sp. 154XXX | 4 | 4 | 5 | 5 | 5 | 5 | 5 | 6 | 0 | | 0% | 0% | 0% | 1.18% |
| 11 | *Archigetes megacephalus* n. sp. XXXXX | 4 | 4 | 5 | 5 | 5 | 5 | 5 | 6 | 0 | 0 | | 0% | 0% | 1.18% |
| 12 | *Archigetes megacephalus* n. sp. MW027493 | 4 | 4 | 5 | 5 | 5 | 5 | 5 | 6 | 0 | 0 | 0 | | 0% | 1.18% |
| 13 | *Archigetes megacephalus* n. sp. MW027494 | 4 | 4 | 5 | 5 | 5 | 5 | 5 | 6 | 0 | 0 | 0 | 0 | | 1.18% |
| 14 | *Archigetes loculotruncatus* n. sp. MW027502 | 18 | 18 | 19 | 18 | 18 | 17 | 18 | 20 | 16 | 16 | 16 | 16 | 16 | |
